# Lipids and α-Synuclein: adding further variables to the equation

**DOI:** 10.3389/fmolb.2024.1455817

**Published:** 2024-08-12

**Authors:** Jana Schepers, Timo Löser, Christian Behl

**Affiliations:** The Autophagy Lab, Institute of Pathobiochemistry, University Medical Center of the Johannes Gutenberg University Mainz, Mainz, Germany

**Keywords:** alpha-Synuclein, Parkinson’s disease, DLB, MSA, membrane lipids, lipid metabolism

## Abstract

Aggregation of alpha-Synuclein (αSyn) has been connected to several neurodegenerative diseases, such as Parkinson’s disease (PD), dementia with Lewy Bodies (DLB), and multiple system atrophy (MSA), that are collected under the umbrella term *synucleinopathies.* The membrane binding abilities of αSyn to negatively charged phospholipids have been well described and are connected to putative physiological functions of αSyn. Consequently, αSyn-related neurodegeneration has been increasingly connected to changes in lipid metabolism and membrane lipid composition. Indeed, αSyn aggregation has been shown to be triggered by the presence of membranes *in vitro*, and some genetic risk factors for PD and DLB are associated with genes coding for proteins directly involved in lipid metabolism. At the same time, αSyn aggregation itself can cause alterations of cellular lipid composition and brain samples of patients also show altered lipid compositions. Thus, it is likely that there is a reciprocal influence between cellular lipid composition and αSyn aggregation, which can be further affected by environmental or genetic factors and ageing. Little is known about lipid changes during physiological ageing and regional differences of the lipid composition of the aged brain. In this review, we aim to summarise our current understanding of lipid changes in connection to αSyn and discuss open questions that need to be answered to further our knowledge of αSyn related neurodegeneration.

## Graphical Abstract

**Figure:**
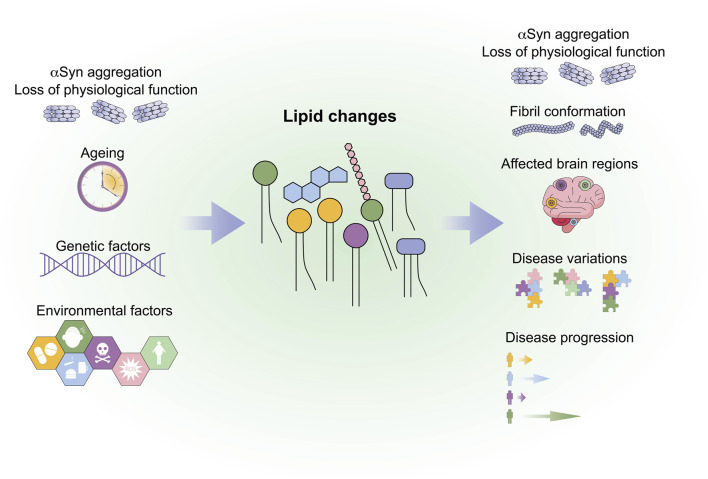
The graphical abstract summarises factors that might lead to lipid changes and possible influences of lipid changes on synucleinopathies.

## 1 Introduction

With approximately 50% of the dry weight of the human brain being lipids, it has one of the highest lipid contents in the human body (Hamilton et al., 2007; Bruce et al., 2017). Strikingly, lipid changes in Alzheimer’s disease (AD) have already been described by Alois Alzheimer upon discovery (Alzheimer et al., 1995) but have not been consequently investigated at that time, perhaps partly due to the lack of adequate methodology. Nowadays, changes in lipid metabolism of the brain are implicated in several neurodegenerative diseases such as, among others, AD, Parkinson’s disease (PD), and amyotrophic lateral sclerosis (ALS) (Wei et al., 2023). For example, it was shown that the lipid metabolism in AD brain tissue is changed, including changes in the fatty acid composition (Nasaruddin et al., 2016; Yin, 2023), accumulation of cholesterol (Xiong et al., 2008; Ahmed et al., 2024), and the presence of the lipoprotein APOE4 isoform as risk factor for AD (Zhu et al., 2015; Lefterov et al., 2019; Miranda et al., 2022; Pires and Rego, 2023; Lozupone and Panza, 2024).

In this review, we focus on α-Synuclein (αSyn) and its growing connection to lipids, not only in the context of its putative physiological functions but also during neurodegenerative processes. Aggregation of αSyn in different neuronal tissues is associated with different neurodegenerative diseases that are collected under the term synucleinopathies. These include, among others, PD, dementia with Lewy Bodies (DLB), and multiple system atrophy (MSA) (Calabresi et al., 2023). To understand disease formation and progression, a lot of successful research has already been conducted, connecting αSyn-aggregation and neurodegeneration to mitochondrial dysfunction and oxidative stress, lysosomal dysfunction, inflammatory processes, and a perturbed Ca^2+^ homeostasis and excitotoxicity (Rocha et al., 2018; Wang et al., 2020; Sahoo et al., 2022; Lyra et al., 2023; Forloni, 2023; Rcom-H’cheo-Gauthier et al., 2016). However, more recently, the link between PD pathogenesis and lipids has gained more and more attention (reviewed in (Alecu and Bennett, 2019; Fanning et al., 2020; Battis et al., 2023; Flores-Leon and Outeiro, 2023)). For example, it was shown that Lewy Bodies (LBs) contain an abundancy of different membranes (Shahmoradian et al., 2019).

We focus on lipid changes and its impact on synucleinopathies, summarising how changes in membrane lipids might contribute to disease progression and whether differences in the membrane composition could contribute to differences in aggregate conformation and localisation. We summarise putative physiological functions of αSyn in connection to membrane lipids as well as lipid-associated processes and discuss lipid changes connected to PD, DLB, and MSA. It is important to keep in mind that, while these three diseases are distinguishable from each other, especially PD and DLB share overlapping disease phenotypes and risk factors (Calabresi et al., 2023). Thus, we compare common factors connecting lipid metabolism that might play a role in all three synucleinopathies but also discuss differences.

As ageing is known to be connected to neurodegenerative synucleinopathies (Poewe et al., 2022; Calabresi et al., 2023), we further discuss current knowledge of changes of the lipid composition of the aged brain. To date, little is known about lipid changes in the brain during physiological ageing even though it might be possible that changes in the regional lipid composition of the brain might explain why different brain regions are affected in different patients. Whether synucleinopathies are induced by age-related lipid changes in the brain remains unclear.

It is known that αSyn forms amyloid fibrils that are rich in β-sheets during pathological processes. During amyloid fibril formation, the structurally disordered αSyn monomers oligomerise to form aggregates that grow into β-sheet rich *protofibrils*. These protofibrils grow to form long amyloid fibrils, that can be detected in LBs (Ghosh et al., 2017; Alam et al., 2019; Mehra et al., 2021). It is known that these amyloid fibrils can adapt different conformations, referred to as *strains* (Bousset et al., 2013). In this review, we address aggregation formation of αSyn in the presence of lipids and discuss how conformational variations of αSyn *strains*, might, to a certain extent, depend on the lipid environment.

To date, there are no disease-modifying treatments available for PD, DLB, and MSA. For PD, the use of L-Dopa to restore dopaminergic function, developed in the 1960s (Cotzias et al., 1967), is still the most common treatment (Fernagut et al., 2014; Stoker and Barker, 2020). Developing alternative treatments and ways to detect pathological events earlier is urgently needed. Thus, understanding the connection between lipid changes, αSyn aggregation, and disease progression has the potential to open new, possible ways of disease-modification and earlier detection.

## 2 Alpha-Synuclein

**FIGURE 1:**
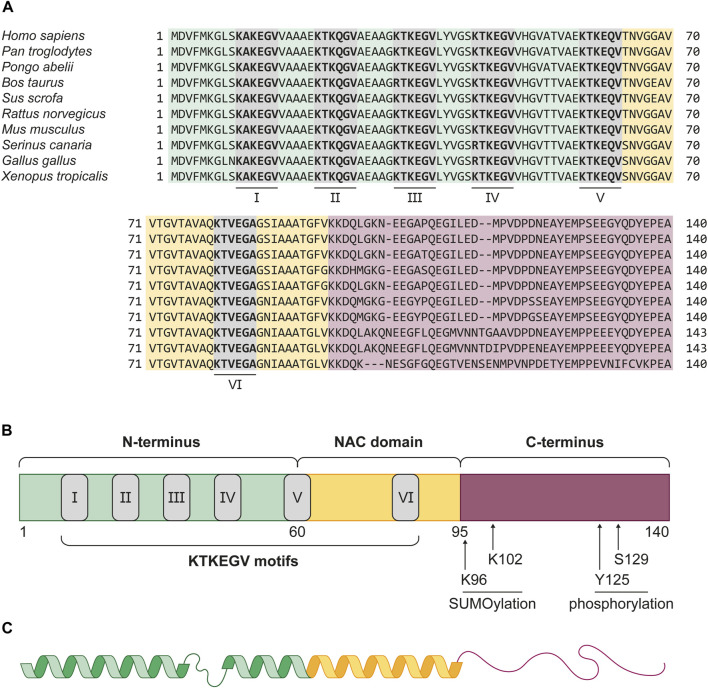
Evolutionary conservation and structure of αSyn. **(A)** Protein sequence alignment of αSyn in vertebrae species *Homo sapiens* (human), *Pan troglodytes* (chimpanzee), *Pongo abelii* (sumatran orangutan), *Bos taurus* (cattle), *Sus scrofa* (wild boar), *Rattus norvegicus* (common rat), *Mus musculus* (house mouse), *Serinus canaria* (atlantic canary), *Gallus gallus* (chicken), and *Xenopus tropicalis* (western clawed frog). The alignment shows a high conservation of the protein across all listed species, especially in the amphipathic N-terminus (green) and NAC domain (yellow). The six KTKEGV motifs (grey boxes), numbered I-VI, show close similarity, with only two differences. In cattle, the first arginine residue in the third motif (III) replaces the chemically very similar lysine residue. The same is observed for the first aa of the fourth KTKEGV motif (IV) in both bird species. In birds, the αSyn protein is also three aa longer than in the other listed organisms. **(B)** αSyn can be structurally divided into the amphipathic N-terminus (green, 1–60 aa), the aggregation promoting NAC-domain (yellow, 61–95 aa) and the acidic C-terminus (red, 96–140 aa) which facilitates protein-protein interactions. The grey boxes depict the KTKEGV motifs, which promote membrane association. At the C-terminus, four post-translational modifications, K96 and K102 SUMOylation, as well as Y125 and S129 phosphorylation, are displayed. These post-translational modifications are suggested to serve physiological functions of αSyn inside the cell. Phosphorylation at S129 is also associated with αSyn pathology. **(C)** Representation αSyn’s secondary structure in the presence of membrane lipids. The N-terminus and NAC domain form an interrupted α-helix which binds to highly curved membranes. The proline-rich C-terminus is considered as intrinsically disordered and flexible.

αSyn is a 14 kDa protein of the small synuclein protein family, which was initially described in the Pacific electric ray *Tetronarce californica* in 1988 and is evolutionary highly conserved (Figure 1A) (Maroteaux et al., 1988; Zhu and Fink, 2003). In humans, the *SNCA* gene, which spans five canonical exons and is located on the PARK1/4 locus of chromosome 4, encodes αSyn. The full-length protein consists of 140 amino acids (aa) and can be divided into three domains (Figure 1B) (Emamzadeh, 2016).

Residues 1–60 form the positively charged N-terminus, which, because of its amphipathic nature, allows interactions with membrane lipids (Bartels et al., 2010; Pirc and Ulrih, 2015). The high amount of lysine residues conducts interactions with anionic lipids, such as phosphatidic acid, phosphatidylinositol, as well as highly negative phosphoinositide phosphates (Middleton and Rhoades, 2010; Jacob et al., 2021a). Upon membrane binding, the coiled N-terminus transforms into a α-helical structure (Figure 1C) (Bussell and Eliezer, 2003; Bodner et al., 2009). Besides an electrostatic interplay, the robustness of this membrane-binding α-helix is strongly dependent on the amount of lipid molecules per protein (Shvadchak et al., 2011; Roeters et al., 2023). The transition from random coil to helix is facilitated by multiple, imperfect repeats of 11 aa, containing a highly conserved KTKEGV motif. Lipid-binding motifs with high similarity were found in apolipoproteins, such as ApoA-I, which also forms α-helices upon membrane binding (Segrest et al., 1990; George et al., 1995). Two of these KTKEGV repeats in αSyn also reach into the second protein domain, the 35 aa long non-amyloid-β component (NAC) (Ueda et al., 1993). In early studies, this domain was found to be prone to aggregation, presumably because of an 11-residue core region, the so-called NACore. Its β-strand structure tends to stack into multiple β-sheets and induces amyloidogenic protein aggregation (Rodriguez et al., 2015; Tuttle et al., 2016; Xu et al., 2016). *In vitro* studies revealed numerous factors that affect the kinetics of αSyn fibril formation. Endogenous factors for αSyn nucleation include protein modifications and truncations, as well as the presence of lipids and membranes (Galvagnion et al., 2015; Ghosh et al., 2017). Environmental factors, such as metals, pesticides, pH, and temperature changes were also found to promote *in vitro* fibrillation (Morris and Finke, 2009; Ghosh et al., 2017). The third protein domain is the anionic C-terminus, which consists of the remaining 46 aa. It is a proline-rich, intrinsically disordered region (random coil) in which around one third of residues are acidic. This comparatively flexible region was found to be a target for multiple post-translational modifications (PTMs), and the central domain for protein-protein interactions (Cole et al., 2002; Oueslati et al., 2010; Manzanza et al., 2021). The majority of investigated PTMs introduced in αSyn were found to inhibit protein function and enhance its susceptibility to pathological aggregation (Zhang et al., 2019). However, recent studies have identified several physiological functions of C-terminal modifications (Figure 1B). For instance, SUMOylation of lysine residues K96 and K102 is required for the nuclear translocation of αSyn (Krumova et al., 2011; Ryu et al., 2019). Additionally, phosphorylation of tyrosine Y125 has been shown to modulate the interaction between αSyn and phospholipase D in human embryonic kidney cells (HEK-293) (Ahn et al., 2002). Recently, it has been shown that phosphorylation at serine S129, which is predominantly related to αSyn pathology, might also regulate αSyn function in healthy cells (Ramalingam et al., 2023).

Exclusively found in vertebrates, αSyn is localised in several different regions of the brain, such as the *substantia nigra*, the cerebral cortex, and hippocampus, among others (Taguchi et al., 2016). Localisation of αSyn in presynaptic terminals as well as its co-localisation with synaptic vesicles (Maroteaux et al., 1988; Bayer et al., 1999; Taguchi et al., 2016) led researchers to believe that αSyn might play an important role in neurotransmission. In the following years, many studies helped elucidate the transport route of αSyn from its synthesis in the cell soma to presynaptic axon terminals and its interactions with different proteins and whole organelles.

## 3 From synthesis to function – interactions of αSyn across the neuron

Since αSyn lacks a canonical translocon sequence, *de novo* biosynthesis of αSyn is most likely directed into the cytoplasm. In the neuronal soma, αSyn is able to interact with a variety of organelles (reviewed in (Bernal-Conde et al., 2019)). Localisation into the nucleus is facilitated via C-terminal SUMOylation and subsequent translocation by karyopherin α6 (Ryu et al., 2019). Because of its small size (<40 kDa), diffusion through the nuclear pore complex inside the nucleus might also be possible (Timney et al., 2016). αSyn can affect DNA persistence length, i.e., physical stiffness, and accessibility for transcription factors, either by direct electrostatic interactions with the DNA backbone, or indirectly, by retaining epigenetic proteins (e.g., histone-modifying enzymes) from entering the nucleus (Desplats et al., 2011; Jiang et al., 2018; Surguchov, 2023). It was observed that these interactions influence DNA condensation through H3K9 methylation and altered histone acetylation (Kontopoulos et al., 2006; Sugeno et al., 2016).

Outside the nucleus, αSyn is found at the outer mitochondrial membrane as well as in mitochondrial sub-compartments (Cole et al., 2008; Devi et al., 2008; Georgas et al., 2009; Menges et al., 2017). At the outer membrane, αSyn suppresses mitochondrial fusion events, which is suggested to benefit the transport of small mitochondrial fragments across the axon (Nakamura et al., 2011; Saxton and Hollenbeck, 2012; Bernal-Conde et al., 2020). However, another study found that drastically changed fusion-fission rates, induced by αSyn overexpression, impair axonal transport (Pozo Devoto et al., 2017). Once inside the mitochondrion, the largest proportion of αSyn accumulates at the inner mitochondrial membrane (IMM), where it most likely interacts with the highly anionic mitochondrial signature phospholipid cardiolipin (Cole et al., 2008; Dudek, 2017; Ryan et al., 2018). This IMM-localisation was mainly found around the electron transport chain (ETC), which is most likely due to its cardiolipin-rich environment (Paradies et al., 2014). The physiological function of αSyn at the ETC is not fully understood, but several lines of evidence suggest that αSyn might stabilise complex I-III electron transfer (Ellis et al., 2005; Devi et al., 2008).

One of the most important processes that αSyn interferes with is vesicular trafficking between the endoplasmic reticulum (ER), the Golgi apparatus (Golgi), and the endosomal shuttle network (Thayanidhi et al., 2010; Teixeira et al., 2021). In several PD models, αSyn was found to interact with membrane fusion factor Rab1 and its homologues, which are associated with ER-Golgi trafficking (Cooper et al., 2006). While the study from Cooper et al. (2006) mainly focused on the detrimental interplay of αSyn with proteins involved in ER-GA transport, previous studies suggested a physiological function of αSyn for *soluble N-ethylmaleimide-sensitive-factor attachment receptor* (SNARE)-dependent membrane fusion events (Burré et al., 2010; Yoo et al., 2023). Accordingly, experiments in *S. cerevisiae* expressing human αSyn showed that the Rab1 homologue Ypt1 colocalises with cytosolic αSyn-accumulations (Soper et al., 2011). Human wild-type αSyn also co-localises with several other yeast Rab proteins, involved in intra-Golgi trafficking, such as Ypt6, Ypt31, and Ypt32 (Soper et al., 2011). In the endo-lysosomal system, αSyn was found to be in close proximity to transport vesicles (Lee et al., 2011; Huang et al., 2019) as well as important factors, such as RAB5A, RAB7, and RAB11A, which play a role in endosomal trafficking (Hasegawa et al., 2011). αSyn being involved in this pathway is supported by the high amounts of anionic, phosphorylated phosphoinositides that comprise endosomal transport vesicles, to which αSyn demonstrates an exceptionally high affinity (Jacob et al., 2021b; Choong et al., 2023).

One of the earliest discovered key functions of αSyn is its involvement in synaptic vesicle trafficking and exocytosis at the pre-synapse (reviewed in (Sharma and Burré, 2023; Nordengen and Morland, 2024)). In fact, the majority of αSyn is found at pre-synaptic axon terminals in adult animals (Maroteaux et al., 1988; Hsu et al., 1998). In order to reach its destination, αSyn is transported along the axon via the slow component b (SCb) (Tang et al., 2012). Besides αSyn, SCb was shown to mainly transport proteins critical for axon growth and regeneration, as well as synaptic function (Roy et al., 2007). Interestingly, the translocation of αSyn to the synapse is also dependent on its association with lipid rafts (Fortin et al., 2004).

At the axon terminal, αSyn was found to play an important, but not essential, role in the life cycle of synaptic vesicles (SV), primarily, but not exclusively, in dopaminergic neurons (reviewed in (Nordengen and Morland, 2024)). During preparation of SV secretion, αSyn participates in different steps, such as monoamine transmitter loading, vesicle docking and - priming (Pifl et al., 2014; Huang et al., 2019). In vesicle priming, αSyn was shown to interact with different proteins that facilitate the fusion of SV and the plasma membrane, e.g., SNARE proteins and the previously mentioned Rab proteins (Burré et al., 2010; Lou et al., 2017). In these processes, the N-terminus of αSyn is proposed to remain in close proximity to the SV membranes, due to its high affinity to anionic and highly curved membranes, while the C-terminus is thought to interact with other proteins (Jensen et al., 1999; Payton et al., 2004; McFarland et al., 2008). Recent data in different cell types show that the localisation of αSyn to the plasma membrane is highly dependent on the abundance of phosphatidylinositol polyphosphates, namely, phosphatidylinositol bisphosphates (PIP2) and phosphatidylinositol trisphosphates (PIP3) (Jacob et al., 2021b). Subsequently, αSyn is also involved in the fusion of SVs and, thereby, the release of neurotransmitters. The presence of αSyn was shown to expand the exocytotic fusion pore at the synapse, favouring full membrane fusion over the faster “kiss-and-run” mechanism (Khounlo et al., 2021). In order to keep the SV pools balanced, αSyn was suggested to aid with endocytosis by introducing higher curvature to the synaptic plasma membrane (Westphal and Chandra, 2013).

**FIGURE 2:**
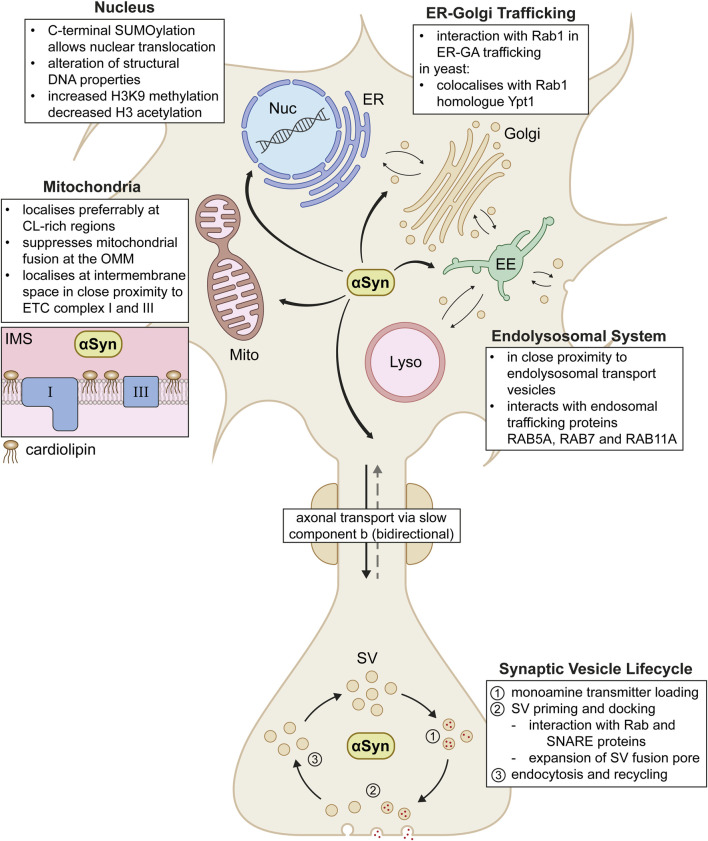
Proposed physiological interactions of αSyn across the neuron. In the cell, αSyn acts in various compartments including the nucleus (Nuc), endoplasmic reticulum (ER), Golgi apparatus (Golgi), early endosomes (EE), lysosomes (Lyso) and mitochondria (Mito). In mitochondria, αSyn localises in the intermembrane space (IMS) in close proximity to electron chain complex I and III (box on the left). αSyn shuttles between soma and synapse via axonal transport via the slow component b. At the synapse, αSyn plays a role in various steps of the entire life cycle of synaptic vesicles (SV). Further interaction details are depicted as text boxes in the figure.

αSyn demonstrates a high variety of localisations and putative physiological functions across the neuron, from soma to axon terminal (Summarised in Figure 2). In PD and LBD, aggregated forms of αSyn that contribute to disease pathology were found to be in close proximity to the beforementioned organelles and pathways (Miraglia et al., 2018; Moors et al., 2021). This underlines the importance of a strict regulation of putative physiological functions of αSyn in affected cellular compartments. Even though interactions with multiple organelles and transport pathways may appear arbitrary at first glance, a shared characteristic unites these diverse localisations of αSyn. αSyn seems to play a key role in general vesicle organisation and membrane fusion events. This was especially observed in highly curved membrane regions rich in anionic phospholipids, such as SVs, general endolysosomal vesicles, or even mitochondria with externalised cardiolipin (Ryan et al., 2018). Given that the above described αSyn-membrane interactions play such a considerable role in αSyn’s putative impact on a variety of neuronal functions, dysregulation of these interactions might be likely to contribute to the generation and/or progression of αSyn related neurodegenerative diseases.

## 4 Synucleinopathies and lipids

### 4.1 PD and lipid changes

The most common synucleinopathy is PD, which is commonly associated with neurodegeneration of dopaminergic neurons in the substantia nigra (SN) and the formation of LBs (Kalia and Lang, 2015). Analyses of patient tissue have revealed that the lipid composition of the brain is changed. These changes include, for example, an increase of diacylglycerols (DAGs) in the frontal cortex of PD patients (Wood et al., 2018). Lipidomic analysis of the visual cortex of PD patients revealed a dramatically altered lipid profile when compared to control brains. These changes include a decrease of unsaturated phosphatidylethanolamine (PE) and differences in the amount of phosphatidylinositol (PI), depending on its fatty acid (FA) chain lengths (Cheng et al., 2011). Similar observations were made when lipidomic analyses were performed upon expression of αSyn in several model systems, where an increase in DAG together with a decrease of several membrane lipid species such as phosphatidylserine (PS) and PI was found (Fanning et al., 2019). Taken together, this suggests that αSyn (-aggregation) may change the lipid composition of the brain.

It is also known that αSyn-lipid interactions depend on the membrane lipid composition. For example, it was shown that increasing the amount of negatively charged gangliosides (GMs) of small unilamellar vesicles (SUVs) *in vitro* increased membrane binding of αSyn (Man et al., 2021). Further *in vitro* studies revealed that the amount of anionic lipids is crucial for αSyn-membrane interactions (Davidson et al., 1998; Andersson et al., 2024) and that more αSyn is able to bind to anionic deformable SUVs, showing that the charge, the flexibility, and the curvature of membranes influence αSyn binding (Andersson et al., 2024; Makasewicz et al., 2024). Furthermore, the interaction of αSyn with membrane lipids was proposed to contribute to aggregate formation (Auluck et al., 2010; Galvagnion et al., 2015). However, other studies have shown that this interaction can prevent αSyn fibril formation (Zhu and Fink, 2003; Martinez et al., 2007). It is important to note that these studies have all been conducted *in vitro* and in correlation with different lipid compositions. While the ratio of PS, phosphatidylcholine (PC), and PE contributed to amyloid aggregation (Galvagnion et al., 2015), interaction with the ganglioside GM1 inhibited it (Martinez et al., 2007). Indeed, GM1 levels were shown to be decreased in brains of PD patients (Hadaczek et al., 2015). Studies on mice deficient for the GM2-synthase, were shown to exhibit PD-like symptoms, which could be alleviated by treatment with LIGA-20, an analogue of GM1 that is able to cross the blood brain barrier (BBB) (Wu et al., 2011). Therefore, the overall lipid composition might not only have a great influence on αSyn membrane interaction but also on αSyn oligomerisation and fibril formation, by either inducing or preventing it. Interestingly, PD-associated mutations of αSyn have been shown to exhibit differential membrane interaction properties (Battis et al., 2023).

Furthermore, some genetic risk factors for PD that are involved in membrane lipid metabolism continue to be identified. One of the most prominent examples are mutations of the *GBA1* gene, coding for the hydrolase glucocerebrosidase (GCase) (Aharon-Peretz et al., 2004; Neumann et al., 2009; Galper et al., 2022; Flores-Leon and Outeiro, 2023). *GBA1* mutations include T369M, T297S, and E326K, among others and cause a reduced activity of the lysosomal GCase (Dos Santos et al., 2024). This is associated with an increased risk for PD (Flores-Leon and Outeiro, 2023; Dos Santos et al., 2024). The GCase hydrolyses glucoceramide to glucose and ceramide in the lysosome and, thus, plays a role in sphingolipid metabolism (Gegg et al., 2022). However, the exact mechanisms of how this leads to PD are still unclear. It is thought that a reduced activity of the GCase inhibits lysosomal function and, thereby, leads to an increased amount of protein aggregation, including aggregation of αSyn (Johnson et al., 2020). It was further shown that αSyn aggregation depends on the FA chain length of GCase substrates; only FA chains longer than C22 induced aggregation (Fredriksen et al., 2021). Conversely, GCase activation enhanced lysosomal activity, which induced clearance of αSyn aggregates (Mazzulli et al., 2016). Interestingly, homozygous mutations of *GBA1* are known to cause Gaucher’s disease (GD), in which symptoms overlap with symptoms known in PD (Johnson et al., 2020).

Another risk factor for PD that is associated with lipid metabolism is Synaptojanin 1 (SYNJ1), which is a PIP-phosphatase (Krebs et al., 2013; Quadri et al., 2013; Olgiati et al., 2014; Ben Romdhan et al., 2018; Schechter and Sharon, 2021). SYNJ1 is part of several pathways involving vesicular structures such as endocytosis (Perera et al., 2006), endosomal trafficking (Watanabe et al., 2018), and autophagy (George et al., 2016; Vanhauwaert et al., 2017). Mutations in SYNJ1’s PIP-phosphatase domain but also other domains are associated with an increased risk for developing PD (Ben Romdhan et al., 2018; Taghavi et al., 2018; Schechter and Sharon, 2021). Again, the exact mechanisms that cause an increased risk for PD are still unclear. It is thought that synaptic dysfunction, caused by SYNJ1 mutations, may trigger neurotoxicity (Brooker et al., 2024). Additionally, mutations of *SH3GL2*, which encodes for the SYNJ1 binding partner endophilin A1, have also been identified as risk factors for PD (Nalls et al., 2019; Brooker et al., 2024). Similarly, mutations in *LRRK2*, a protein kinase that phosphorylates SYNJ1 and is involved in endocytosis (Pan et al., 2017; Schechter and Sharon, 2021) and autophagy (reviewed in (Madureira et al., 2020)) have been identified as risk factors for PD (Summarised in Table 1). Again, detailed molecular mechanisms remain unclear. Taken together, dysregulation of lipid homeostasis, whether directly or indirectly, is likely to affect cellular function and, thus, contributes to PD formation and/or progression.

**TABLE 1:** Summary of lipid-related connections to αSyn pathology in PD, DLB, and MSA.

α-Synucleinopathy	Affected brain region	Connection to lipids (including genetic factors)	Aggregates found	References
Parkinson’s Disease (PD)	• Dopaminergic neurons of the substantia nigra (pars compacta)	*Patient data* • Increase of DAG in the frontal cortex of PD patients • General change of the lipid profile of the visual cortex of PD patients including a decrease of unsaturated PE and FA-chain length dependent changes in the amounts of PI • Decrease of GM1 in brains of PD patients *In vitro studies* •αSyn has a higher binding affinity to negatively charged/anionic membrane lipids and to vesicular membranes • PS, PC, and PE to αSyn ratio contributes to amyloid aggregation • GM1 inhibits amyloid aggregation *In vivo studies* • Increase of DAG, decrease of PS and PI • GM2-synthase deficient mice show PD-like symptoms, which can be alleviated by GM1-analogue treatment *Genetic risk factors* • Mutations of *GBA1* • Mutations of *SH3GL2* • Mutations of *LRRK2*	• Formation of Lewy Bodies (LBs) and Lewy Neurites • Aggregates contain a Lewy fold: three layered aggregates comprised of residues 31–100 of αSyn that form a total of 9 β-sheet strands	Davidson et al. (1998), Aharon-Peretz et al. (2004), Martinez et al. (2007), Neumann et al. (2009), Cheng et al. (2011), Wu et al. (2011), Krebs et al. (2013), Quadri et al. (2013), Olgiati et al. (2014), Galvagnion et al. (2015), Hadaczek et al. (2015), Kalia and Lang (2015), Ben Romdhan et al. (2018), Wood et al. (2018), Fanning et al. (2019), Johnson et al. (2020), Man et al. (2021), Schechter and Sharon (2021), Galper et al. (2022), Gegg et al. (2022), Yang et al. (2022), Flores-Leon and Outeiro (2023), Andersson et al. (2024), Dos Santos et al. (2024), Makasewicz et al. (2024)
Dementia with Lewy Bodies (DLB)	• Neocortex • Limbic system • Dopaminergic neurons of the substantia nigra (pars compacta)	*Patient data* • Decrease of several phospholipids in brains of *APOE*ε4 carriers (in the context of AD) • In the context of AD: reduced levels of PIP2 *In vivo studies* • In the context of APOE ε4 KI mice: reduced levels of PIP2 and reduced degradation of *SYNJ1*mRNA *Genetic risk factors* • Mutations of *GBA1* • Carriers of *APOE*ε*4*	• Formation of LBs and LNs • Aggregates contain a Lewy fold: three layered aggregates comprised of residues 31–100 of αSyn that form a total of 9 β-sheet strands	Nalls et al. (2013), Zhu et al. (2015), Lefterov et al. (2019), Outeiro et al. (2019), Lee et al. (2021), Yang et al. (2022)
Multiple System Atrophy (MSA)	• MSA-P (with parkinsonism): midbrain and basal ganglia • MSA-C (with cerebral ataxia): midbrain, cerebellum, and brainstem	*Patient data* • Low serum levels of cholesterol, LDL-C, HDL-C (lower in MSA-C patients), and TG are associated with both, MSA-C and MSA-P, but have no effect on disease progression *In vivo studies* • Transcriptome analysis of striatal astrocytes of a MSA mouse model revealed a downregulation of genes involved in lipid metabolism *Genetic risk factors* • inconclusive • weak connection to APOE ε4	• Formation of glial cytoplasmic inclusions (GCIs) in oligodendrocytes • Aggregates form asymmetrical Type I or Type II filaments • Type I filaments are made of two protofibrils: PF-1A is formed by residues 14–94 and contains 12 β-sheets and PF-1B is formed by residues 21–99 and contains ten β-sheets • Type II filaments are made of two protofibrils: PF-IIA is formed by residues 14–94 and also contains 12 β-sheets but has a different conformation to PF-IA. PF-IIB is formed by residues 36–99 and contains nine β-sheets	Lee et al. (2009), Cao et al. (2014), Robinson et al. (2018), Schweighauser et al. (2020), Poewe et al. (2022), So and Watts (2023), Schneider et al. (2024)

As most data on αSyn and lipid homeostasis exist in the context of PD, little is known about lipid changes, maybe even in other brain regions, that may also be altered in other synucleinopathies. The question here is whether similar changes in lipid composition might be a common factor in all synucleinopathies and whether changes occur in different regions of the brain, which might explain the differences between the synucleinopathies. Lastly, whether and how the lipid composition influences aggregate conformation known to vary in different synucleinopathies still needs to be investigated.

### 4.2 DLB and lipid changes

Formation of LBs and a loss of dopaminergic neurons of the SN, together with a reduction of cortical neurons and neurons of the limbic system, are commonly associated with DLB (Outeiro et al., 2019). In DLB, LBs can also be found in different regions of the brain besides the SN, such as the neocortex and the limbic system (Outeiro et al., 2019). There is still very little data connecting changes in lipid homeostasis to DLB but a few genetic risk factors are known that overlap with risk factors for PD.

The most common genetic risk factors for DLB, shared with PD, are mutations of *GBA1*, causing changes in the functionality of the GCase, a dysregulation of sphingolipid metabolism, and changes in autophagy function (see above) (Lee et al., 2021). It was suggested that mutations of *GBA1* may even have a stronger association to DLB than to PD (Nalls et al., 2013; Lee et al., 2021). Another risk factor involved in lipid homeostasis that is associated with DLB is the presence of the *APOE ε4* isoform of the apolipoprotein E (APOE) (Tsuang et al., 2013; Bras et al., 2014). APOE has three isoforms (ε2, ε3, and ε4) and is mainly expressed in astrocytes. It plays a role in cholesterol and lipid transport across the brain, which is important for neuronal function (Yamazaki et al., 2019; Jin et al., 2022). Interestingly, the presence of the *APOE ε*4 isoform has also been associated with Alzheimer’s Disease (AD) (Lee et al., 2021; Pires and Rego, 2023; Fortea et al., 2024; Lozupone and Panza, 2024). An inefficient lipid transport from astrocytes to neurons is known to change neuronal lipid composition (Lefterov et al., 2019; Miranda et al., 2022). Interestingly, it was found in the context of AD, that carriers of the *APOE ε4* allele have reduced levels of PIP2, which was explained by a decreased degradation of the *SYNJ1* mRNA (Zhu et al., 2015). As mentioned above, mutations in *SYNJ1* itself are known risk factors for PD (Krebs et al., 2013; Quadri et al., 2013; Olgiati et al., 2014; Ben Romdhan et al., 2018; Schechter and Sharon, 2021), however, whether this is also the case for DLB is still unclear. Further research into a possible connection of *SYNJ1* mutations and DLB would help to clarify this. Maybe unsurprisingly, DLB is often not clearly distinguishable from AD (dementia only) or PD (Parkinsonism with dementia) (Noe et al., 2004; Jellinger and Korczyn, 2018; Nedelec et al., 2023) (Summarised in Table 1).

In general, the presence of αSyn itself is already changing the cellular lipid composition (see above), and, thus, it might be likely that this is also the case in other synucleinopathies. The combination of genetic factors that change the cellular lipid profile might be one of the factors leading to or accelerating disease progression.

### 4.3 MSA and lipid changes

MSA is a rare neurodegenerative synucleinopathy and, in contrast to PD and DLB, associated with the formation of αSyn aggregates in oligodendrocytes referred to as glial cytoplasmic inclusions (GCIs) (Spillantini et al., 1998; Poewe et al., 2022). One of the hallmarks of MSA is a demyelination of neurons, which is connected to GCI-formation (Poewe et al., 2022). Myelin, multiple layers of membranes looped around the axon, contains a higher proportion of cholesterol and glycolipids (e.g., glycosylceramide) than other cellular membranes (Baumann and Pham-Dinh, 2001; Poitelon et al., 2020).

Little is known about the connections between GCI formation and the unique lipid composition of myelin sheaths in the context of MSA. It was shown that lower cholesterol levels and lower levels of LDL-C and HDL-C in patient serum have been connected to an increased risk of developing MSA (Lee et al., 2009; Cao et al., 2014). However, it is known that lipoproteins carrying cholesterol outside the central nervous system do not cross the blood brain barrier (BBB) and that cholesterol in the brain is mainly synthesised in astrocytes (Bleasel et al., 2014; Pifferi et al., 2021; Li et al., 2022). Thus, the connections between serum cholesterol levels and lipid changes in the brain during MSA remain elusive on a molecular level. In an MSA mouse model, transcriptome analyses of astrocytes implicated a dysregulation of cellular lipid metabolism (Schneider et al., 2024). This might point towards a changed lipid homeostasis in MSA but needs to be investigated more thoroughly.

Genetic risk factors for MSA that connect to lipid homeostasis are currently unknown. There are inconclusive studies on the genetic background of MSA (Poewe et al., 2022). Interestingly, one study suggests that the frequency of MSA-patients carrying the *APOE ε2* isoform is lower than the frequency of MSA patients carrying the *APOE ε4* isoform (Robinson et al., 2018) (Summarised in Table 1).

Taken together, MSA remains the rarest and, in terms of connection to lipids, the most elusive synucleinopathy, mostly due to the inconclusive evidence for a genetic background. Nevertheless, more research effort has to be directed towards understanding the differences or similarities between PD, DLB, and MSA.

## 5 Lipid changes in physiological ageing

Given that ageing is one of the biggest risk factors for developing neurodegenerative diseases such as PD, and that most neurodegenerative diseases occur sporadically, it is of great interest to understand lipid changes in the aged brain. While there has been a lot of research effort to better understand disease-related lipid changes in the brain, less is known about the possible lipid changes during physiological ageing. Very early studies analysing whole brains have described a general decline of total lipids with age (Rouser and Yamamoto, 1968; Mesa-Herrera et al., 2019). Later, analyses of white matter and cerebral cortices of the temporal and frontal lobes confirmed these findings (Svennerholm et al., 1991). More specific analyses of lipid classes revealed, for example, a reduction of polyunsaturated fatty acids (PUFAs) in the orbitofrontal cortex with age (McNamara et al., 2008). A more recent study has found that, while the overall lipid concentrations in the prefrontal cortex remain at a similar level with age, the lipid profile itself undergoes changes with a transition point of about 50–55 years of age. Some affected pathways were shown to be unsaturated fatty acid biosynthesis and glycerolipid metabolism, with differences between males and females (Yu et al., 2020). Interestingly, regional lipid profile diversity was also shown to change with age (Mota-Martorell et al., 2022). However, given the complexity of the brain, lipid changes in the physiologically ageing brain are still not clearly understood. Being able to differentiate between changes in healthy ageing and changes that might be part of, or even precede, pathological processes of neurodegeneration is of great importance to prevent and/or treat these diseases.

In an effort to find potential disease markers for PD, a significant amount of research has been focusing on lipidomic analyses of patient serum. For example, serum analyses of patients carrying the A53T mutation of *SNCA* revealed an increase of diacylglycerol, triacylglycerol, and PC (Avisar et al., 2022). Similarly, a decrease of serum levels of HDL-C was found in patients with PD (Choe et al., 2021). Furthermore, patients carrying a mutation in *LRRK2* showed changes in ceramide (Cer), TAG, sphingomyelin, PC, and lyso-phosphatidylethanolamine (LPE) (Galper et al., 2022). Analysis of samples from patients with idiopathic PD showed similar findings with lower levels of PS, some Cer species, and Sphingomyelin (SM) (Dahabiyeh et al., 2023). While these findings might pave the path to potential serum markers for disease, this is only the beginning of more extensive research to come. The challenge here is to find common markers that are reliably enough for all variants of PD, as it is a disease caused by multiple factors, many of which have not yet been completely understood.

## 6 Lipid interactions and possible influences on aggregate formation

Interestingly, it is known that different synucleinopathies exhibit different fibrillar αSyn conformations. These conformational differences are referred to as αSyn strains and, similarly to what is already known in prion diseases, they show different characteristics in terms of disease progression (Prusiner, 2012; Bousset et al., 2013; Peng et al., 2018; Woerman et al., 2019). When comparing LBs to GCIs from MSA patients, for example, the conformation of the accumulations was shown to be clearly distinct from each other (Peng et al., 2018; Shahnawaz et al., 2020). Indeed, two types of filaments were found in MSA patient brain extracts: Type I filaments were larger and showed a distinct folding when compared to the smaller Type II filaments. Both filaments were found to be asymmetrical and made of two protofilaments each. These protofilaments contain between 9 and 12 β-sheets (Schweighauser et al., 2020; So and Watts, 2023). αSyn filaments derived from patients with DLB or PD, on the other hand, showed identical conformations containing an ordered core called the *Lewy fold*, which is a three-layered aggregate with a total of nine β-sheets formed by residues 31–100 (Table 1) (Yang et al., 2022). *In vitro*, recombinant αSyn showed a larger variation in aggregate conformation, depending on chemical conditions under which the aggregations were formed (So and Watts, 2023). These variants exhibited different effectivities of prion-like seeding properties (Walker and Jucker, 2015; Goedert et al., 2017), e.g., the propagation from cell to cell within the brain (Torre-Muruzabal et al., 2023).

However, the reason for these conformational differences that cause different disease phenotypes in synucleinopathies are not well understood. It is known that αSyn aggregation can be triggered by interaction with lipids (Makasewicz et al., 2024). Using *in vitro* membrane models including small unilamellar vesicles (SUVs), giant unilamellar vesicles (GUVs), and flat supported lipid bilayers, it was shown that lipid interaction of αSyn can induce nucleation of aggregates (Grey et al., 2011; Galvagnion et al., 2015; Makasewicz et al., 2021; Dear et al., 2024). These processes are dependent on the lipid composition of the vesicular structures investigated, the amount of negatively charged lipids, membrane fluidity, and membrane curvature (reviewed in (Makasewicz et al., 2024)).

Furthermore, familial variants of αSyn are found to be N-terminally acetylated in LBs (Anderson et al., 2006). Recently, it was shown that N-terminal acetylation of familial variants of αSyn can change the structure of the fibrillar aggregates and the lipid binding properties individually for each investigated variant (Bell et al., 2023). These findings point towards highly complex processes involved in the formation of synucleinopathies, implicating, among others, lipid composition, post-translational modifications, and possible mutations of *SNCA*.

Based on this, it might not be unlikely that changes in cellular lipid composition, occurring with age, through mutations in genes involved in lipid homeostasis, or through individual lifestyle and environmental factors, influence disease onset, variation, severity, and progression. Thinking a little further, this might even mean that differences of lipid compositions within a single brain (Mota-Martorell et al., 2022) could explain regional specificity of protein aggregates and symptom-phenotype variations. Indeed, it was recently found that distinct aggregate variants can be found in different brain regions (Wiseman et al., 2024). Taken together, understanding changes in the lipid composition of different brain regions and how this affects disease is likely to be one of the significant steps towards understanding the progression and onset of synucleinopathies. An improved understanding of the underlying processes will open new paths towards treatment or even disease prevention.

## 7 Discussion

The putative physiological functions and membrane binding properties of αSyn and processes during neurodegeneration are strongly connected to lipid changes in the brain. While synucleinopathies are all known to be multifactorial neurodegenerative diseases, it is possible that some of the factors currently recognised as contributing to disease development and progression might be rooted in changes in lipid metabolism or membrane lipid composition (Reviewed in (Flores-Leon and Outeiro, 2023)). For example, it was shown that a lack of the well-established risk factor for PD *PINK1* causes an accumulation of ceramides in the mitochondrial membrane, inhibiting β-oxidation and causing degradation of mitochondria via mitophagy (Vos et al., 2021; Flores-Leon and Outeiro, 2023). Even though the exact physiological roles of αSyn still remain to be determined, much progress has been made. With the help of advanced analytical methods, understanding the connection between lipid changes (storage, metabolism, lipid rafts, membrane composition) and αSyn is of importance to provide a deeper insight into the ever-increasing complexity of synucleinopathies.

Individual genetic risk factors, environmental and nutritional factors, and ageing, might all have an impact on the lipid composition of the brain. To date, very little is known about lipid-changes during physiological ageing although understanding these processes might be key to develop new research approaches for prevention or treatment of synucleinopathies. Furthermore, being able to distinguish between physiological lipid changes and alterations that contribute to disease progression will contribute substantially to future research of neurodegeneration. More progress is needed to understand regional lipid changes and their potential impact on disease development. Considering that these changes may result from a combination of several factors that are likely to be individual for each affected person, personalised assessments and treatments should be considered in the future. If different lipid compositions affect aggregate conformation and, with that, influence the rate of disease progression and spread throughout the brain, it might potentially open new ways of disease prevention or inspire novel therapeutical approaches.

A lot of research has already been conducted on future therapeutical or preventative treatments. One approach, for example, is the use of lipidic nanoparticles for drug delivery (Tsakiri et al., 2024), which could be adapted for targeting lipid changes in the brain, a concept referred to as *membrane lipid therapy* (Escriba et al., 2015). However, for that, we require a deeper understanding of the molecular mechanisms of lipid changes in synucleinopathies. Other major challenges that need to be addressed are ways to diagnose and classify neurodegenerative diseases such as synucleinopathies earlier and before the onset of clinical symptoms. Here, we can expand on the research efforts into finding reliable early biomarkers for PD such as, for example, αSyn seeding assays of cerebrospinal fluid (Orru et al., 2021; Rutledge et al., 2024).

Although a lot of factors connected to disease formation are already well understood, the influence of lipids on these processes have only recently gained more attention. Taken together, future research efforts should be made to (i) better understand differences between lipid changes that occur during physiological aging and lipid changes associated with pathological processes; (ii) to understand how regional differences in the lipid composition might contribute to aggregate localisation and conformation and, with that, influence the speed of disease progression and symptom variations; (iii) and to find reliable markers that can detect pathological processes earlier. Viewing synucleinopathies through the lens of lipid alterations alongside other well-established disease contributors possibly holds the potential to find novel approaches in disease diagnosis and therapy.

## References

[B1] Aharon-Peretz J. Rosenbaum H. Gershoni-Baruch R. (2004). Mutations in the glucocerebrosidase gene and Parkinson's disease in Ashkenazi Jews. N. Engl. J. Med. 351, 1972–1977. 10.1056/NEJMoa033277 15525722

[B2] Ahmed H. Wang Y. Griffiths W. J. Levey A. I. Pikuleva I. Liang S. H. (2024). Brain cholesterol and Alzheimer's disease: challenges and opportunities in probe and drug development. Brain 147, 1622–1635. 10.1093/brain/awae028 38301270 PMC11068113

[B3] Ahn B. H. Rhim H. Kim S. Y. Sung Y. M. Lee M. Y. Choi J. Y. (2002). alpha-Synuclein interacts with phospholipase D isozymes and inhibits pervanadate-induced phospholipase D activation in human embryonic kidney-293 cells. J. Biol. Chem. 277, 12334–12342. 10.1074/jbc.M110414200 11821392

[B4] Alam P. Bousset L. Melki R. Otzen D. E. (2019). α-synuclein oligomers and fibrils: a spectrum of species, a spectrum of toxicities. J. Neurochem. 150, 522–534. 10.1111/jnc.14808 31254394

[B5] Alecu I. Bennett S. A. L. (2019). Dysregulated lipid metabolism and its role in α-synucleinopathy in Parkinson's disease. Front. Neurosci. 13, 328. 10.3389/fnins.2019.00328 31031582 PMC6470291

[B6] Alzheimer A. Stelzmann R. A. Schnitzlein H. N. Murtagh F. R. (1995). An English translation of Alzheimer's 1907 paper, “Uber eine eigenartige Erkankung der Hirnrinde. Clin. Anat. 8, 429–431. 10.1002/ca.980080612 8713166

[B7] Anderson J. P. Walker D. E. Goldstein J. M. de Laat R. Banducci K. Caccavello R. J. (2006). Phosphorylation of Ser-129 is the dominant pathological modification of alpha-synuclein in familial and sporadic Lewy body disease. J. Biol. Chem. 281, 29739–29752. 10.1074/jbc.M600933200 16847063

[B8] Andersson A. Linse S. Sparr E. Fornasier M. Jonsson P. (2024). The density of anionic lipids modulates the adsorption of α-Synuclein onto lipid membranes. Biophys. Chem. 305, 107143. 10.1016/j.bpc.2023.107143 38100855

[B9] Auluck P. K. Caraveo G. Lindquist S. (2010). α-Synuclein: membrane interactions and toxicity in Parkinson's disease. Annu. Rev. Cell Dev. Biol. 26, 211–233. 10.1146/annurev.cellbio.042308.113313 20500090

[B10] Avisar H. Guardia-Laguarta C. Surface M. Papagiannakis N. Maniati M. Antonellou R. (2022). Lipid level alteration in human and cellular models of alpha synuclein mutations. NPJ Park. Dis. 8, 52. 10.1038/s41531-022-00313-y PMC9039073 35468903

[B11] Bartels T. Ahlstrom L. S. Leftin A. Kamp F. Haass C. Brown M. F. (2010). The N-terminus of the intrinsically disordered protein α-synuclein triggers membrane binding and helix folding. Biophys. J. 99, 2116–2124. 10.1016/j.bpj.2010.06.035 20923645 PMC3042581

[B12] Battis K. Xiang W. Winkler J. (2023). The bidirectional interplay of a-synuclein with lipids in the central nervous system and its implications for the pathogenesis of Parkinson's disease. Int. J. Mol. Sci. 24, 13270. 10.3390/ijms241713270 37686080 PMC10487772

[B13] Baumann N. Pham-Dinh D. (2001). Biology of oligodendrocyte and myelin in the mammalian central nervous system. Physiol. Rev. 81, 871–927. 10.1152/physrev.2001.81.2.871 11274346

[B14] Bayer T. A. Jakala P. Hartmann T. Egensperger R. Buslei R. Falkai P. (1999). Neural expression profile of alpha-synuclein in developing human cortex. Neuroreport 10, 2799–2803. 10.1097/00001756-199909090-00019 10511443

[B15] Bell R. Castellana-Cruz M. Nene A. Thrush R. J. Xu C. K. Kumita J. R. (2023). Effects of N-terminal acetylation on the aggregation of disease-related α-synuclein variants. J. Mol. Biol. 435, 167825. 10.1016/j.jmb.2022.167825 36099961

[B16] Ben Romdhan S. Sakka S. Farhat N. Triki S. Dammak M. Mhiri C. (2018). A novel SYNJ1 mutation in a Tunisian family with Juvenile Parkinson's disease associated with epilepsy. J. Mol. Neurosci. 66, 273–278. 10.1007/s12031-018-1167-2 30187305

[B17] Bernal-Conde L. D. Ramos-Acevedo R. Reyes-Hernandez M. A. Balbuena-Olvera A. J. Morales-Moreno I. D. Arguero-Sanchez R. (2019). Alpha-synuclein physiology and pathology: a perspective on cellular structures and organelles. Front. Neurosci. 13, 1399. 10.3389/fnins.2019.01399 32038126 PMC6989544

[B18] Bernal-Conde L. D. Ramos-Acevedo R. Reyes-Hernández M. A. Balbuena-Olvera A. J. Morales-Moreno I. D. Argüero-Sánchez R. (2020). Alpha-synuclein physiology and pathology: a perspective on cellular structures and organelles. Front. Neurosci. 13. 10.3389/fnins.2019.01399 PMC6989544 32038126

[B19] Bleasel J. M. Wong J. H. Halliday G. M. Kim W. S. (2014). Lipid dysfunction and pathogenesis of multiple system atrophy. Acta Neuropathol. Commun. 2, 15. 10.1186/2051-5960-2-15 24502382 PMC3922275

[B20] Bodner C. R. Dobson C. M. Bax A. (2009). Multiple tight phospholipid-binding modes of alpha-synuclein revealed by solution NMR spectroscopy. J. Mol. Biol. 390, 775–790. 10.1016/j.jmb.2009.05.066 19481095 PMC2709488

[B21] Bousset L. Pieri L. Ruiz-Arlandis G. Gath J. Jensen P. H. Habenstein B. (2013). Structural and functional characterization of two alpha-synuclein strains. Nat. Commun. 4, 2575. 10.1038/ncomms3575 24108358 PMC3826637

[B22] Bras J. Guerreiro R. Darwent L. Parkkinen L. Ansorge O. Escott-Price V. (2014). Genetic analysis implicates APOE, SNCA and suggests lysosomal dysfunction in the etiology of dementia with Lewy bodies. Hum. Mol. Genet. 23, 6139–6146. 10.1093/hmg/ddu334 24973356 PMC4222357

[B23] Brooker S. M. Naylor G. E. Krainc D. (2024). Cell biology of Parkinson's disease: mechanisms of synaptic, lysosomal, and mitochondrial dysfunction. Curr. Opin. Neurobiol. 85, 102841. 10.1016/j.conb.2024.102841 38306948 PMC12110379

[B24] Bruce K. D. Zsombok A. Eckel R. H. (2017). Lipid processing in the brain: a key regulator of systemic metabolism. Front. Endocrinol. (Lausanne) 8, 60. 10.3389/fendo.2017.00060 28421037 PMC5378716

[B25] Burré J. Sharma M. Tsetsenis T. Buchman V. Etherton M. R. Südhof T. C. (2010). Alpha-synuclein promotes SNARE-complex assembly *in vivo* and *in vitro* . Science 329, 1663–1667. 10.1126/science.1195227 20798282 PMC3235365

[B26] Bussell R. Eliezer D. (2003). A structural and functional role for 11-mer repeats in α-synuclein and other exchangeable lipid binding proteins. J. Mol. Biol. 329, 763–778. 10.1016/s0022-2836(03)00520-5 12787676

[B27] Calabresi P. Mechelli A. Natale G. Volpicelli-Daley L. Di Lazzaro G. Ghiglieri V. (2023). Alpha-synuclein in Parkinson's disease and other synucleinopathies: from overt neurodegeneration back to early synaptic dysfunction. Cell death Dis. 14, 176. 10.1038/s41419-023-05672-9 36859484 PMC9977911

[B28] Cao B. Guo X. Chen K. Song W. Huang R. Wei Q. Q. (2014). Serum lipid levels are associated with the prevalence but not with the disease progression of multiple system atrophy in a Chinese population. Neurol. Res. 36, 150–156. 10.1179/1743132813y.0000000277 24172715

[B29] Cheng D. Jenner A. M. Shui G. Cheong W. F. Mitchell T. W. Nealon J. R. (2011). Lipid pathway alterations in Parkinson's disease primary visual cortex. PloS one 6, e17299. 10.1371/journal.pone.0017299 21387008 PMC3046155

[B30] Choe C. U. Petersen E. Lezius S. Cheng B. Schulz R. Buhmann C. (2021). Association of lipid levels with motor and cognitive function and decline in advanced Parkinson's disease in the Mark-PD study. Park. Relat. Disord. 85, 5–10. 10.1016/j.parkreldis.2021.02.007 33636481

[B31] Choong C.-J. Aguirre C. Kakuda K. Beck G. Nakanishi H. Kimura Y. (2023). Phosphatidylinositol-3, 4, 5-trisphosphate interacts with alpha-synuclein and initiates its aggregation and formation of Parkinson’s disease-related fibril polymorphism. Acta Neuropathol. 145, 573–595. 10.1007/s00401-023-02555-3 36939875 PMC10119223

[B32] Cole N. B. DiEuliis D. Leo P. Mitchell D. C. Nussbaum R. L. (2008). Mitochondrial translocation of alpha-synuclein is promoted by intracellular acidification. Exp. Cell Res. 314, 2076–2089. 10.1016/j.yexcr.2008.03.012 18440504 PMC2483835

[B33] Cole N. B. Murphy D. D. Grider T. Rueter S. Brasaemle D. Nussbaum R. L. (2002). Lipid droplet binding and oligomerization properties of the Parkinson's disease protein alpha-synuclein. J. Biol. Chem. 277, 6344–6352. 10.1074/jbc.M108414200 11744721

[B34] Cooper A. A. Gitler A. D. Cashikar A. Haynes C. M. Hill K. J. Bhullar B. (2006). Alpha-synuclein blocks ER-golgi traffic and Rab1 rescues neuron loss in Parkinson's models. Science 313, 324–328. 10.1126/science.1129462 16794039 PMC1983366

[B35] Cotzias G. C. Van Woert M. H. Schiffer L. M. (1967). Aromatic amino acids and modification of parkinsonism. N. Engl. J. Med. 276, 374–379. 10.1056/nejm196702162760703 5334614

[B36] Dahabiyeh L. A. Nimer R. M. Rashed M. Wells J. D. Fiehn O. (2023). Serum-based lipid panels for diagnosis of idiopathic Parkinson's disease. Metabolites 13, 990. 10.3390/metabo13090990 37755270 PMC10537766

[B37] Davidson W. S. Jonas A. Clayton D. F. George J. M. (1998). Stabilization of alpha-synuclein secondary structure upon binding to synthetic membranes. J. Biol. Chem. 273, 9443–9449. 10.1074/jbc.273.16.9443 9545270

[B38] Dear A. J. Teng X. Ball S. R. Lewin J. Horne R. I. Clow D. (2024). Molecular mechanism of α-synuclein aggregation on lipid membranes revealed. Chem. Sci. 15, 7229–7242. 10.1039/d3sc05661a 38756798 PMC11095391

[B39] Desplats P. Spencer B. Coffee E. Patel P. Michael S. Patrick C. (2011). Alpha-synuclein sequesters Dnmt1 from the nucleus: a novel mechanism for epigenetic alterations in Lewy body diseases. J. Biol. Chem. 286, 9031–9037. 10.1074/jbc.C110.212589 21296890 PMC3059002

[B40] Devi L. Raghavendran V. Prabhu B. M. Avadhani N. G. Anandatheerthavarada H. K. (2008). Mitochondrial import and accumulation of alpha-synuclein impair complex I in human dopaminergic neuronal cultures and Parkinson disease brain. J. Biol. Chem. 283, 9089–9100. 10.1074/jbc.M710012200 18245082 PMC2431021

[B41] Dos Santos J. C. C. Mano G. B. C. da Cunha Barreto-Vianna A. R. Garcia T. F. M. de Vasconcelos A. V. Sa C. S. G. (2024). The molecular impact of glucosylceramidase beta 1 (Gba1) in Parkinson's disease: a new genetic state of the art. Mol. Neurobiol. 10.1007/s12035-024-04008-8 38347286

[B42] Dudek J. (2017). Role of cardiolipin in mitochondrial signaling pathways. Front. Cell Dev. Biol. 5, 90. 10.3389/fcell.2017.00090 29034233 PMC5626828

[B43] Ellis C. E. Murphy E. J. Mitchell D. C. Golovko M. Y. Scaglia F. Barceló-Coblijn G. C. (2005). Mitochondrial lipid abnormality and electron transport chain impairment in mice lacking α-synuclein. Mol. Cell. Biol. 25, 10190–10201. 10.1128/mcb.25.22.10190-10201.2005 16260631 PMC1280279

[B44] Emamzadeh F. N. (2016). Alpha-synuclein structure, functions, and interactions. J. Res. Med. Sci. 21, 29. 10.4103/1735-1995.181989 27904575 PMC5122110

[B45] Escriba P. V. Busquets X. Inokuchi J. Balogh G. Torok Z. Horvath I. (2015). Membrane lipid therapy: modulation of the cell membrane composition and structure as a molecular base for drug discovery and new disease treatment. Prog. Lipid Res. 59, 38–53. 10.1016/j.plipres.2015.04.003 25969421

[B46] Fanning S. Haque A. Imberdis T. Baru V. Barrasa M. I. Nuber S. (2019). Lipidomic analysis of α-synuclein neurotoxicity identifies stearoyl CoA desaturase as a target for Parkinson treatment. Mol. Cell 73, 1001–1014 e8. 10.1016/j.molcel.2018.11.028 30527540 PMC6408259

[B47] Fanning S. Selkoe D. Dettmer U. (2020). Parkinson's disease: proteinopathy or lipidopathy? NPJ Park. Dis. 6, 3. 10.1038/s41531-019-0103-7 PMC6941970 31909184

[B48] Fernagut P. O. Dehay B. Maillard A. Bezard E. Perez P. Pavy-Le Traon A. (2014). Multiple system atrophy: a prototypical synucleinopathy for disease-modifying therapeutic strategies. Neurobiol. Dis. 67, 133–139. 10.1016/j.nbd.2014.03.021 24727096

[B49] Flores-Leon M. Outeiro T. F. (2023). More than meets the eye in Parkinson's disease and other synucleinopathies: from proteinopathy to lipidopathy. Acta Neuropathol. 146, 369–385. 10.1007/s00401-023-02601-0 37421475 PMC10412683

[B50] Forloni G. (2023). Alpha synuclein: neurodegeneration and inflammation. Int. J. Mol. Sci. 24, 5914. 10.3390/ijms24065914 36982988 PMC10059798

[B51] Fortea J. Pegueroles J. Alcolea D. Belbin O. Dols-Icardo O. Vaqué-Alcázar L. (2024). APOE4 homozygozity represents a distinct genetic form of Alzheimer's disease. Nat. Med. 30, 1284–1291. 10.1038/s41591-024-02931-w 38710950 PMC13310155

[B52] Fortin D. L. Troyer M. D. Nakamura K. Kubo S. Anthony M. D. Edwards R. H. (2004). Lipid rafts mediate the synaptic localization of alpha-synuclein. J. Neurosci. official J. Soc. Neurosci. 24, 6715–6723. 10.1523/JNEUROSCI.1594-04.2004 PMC6729723 15282274

[B53] Fredriksen K. Aivazidis S. Sharma K. Burbidge K. J. Pitcairn C. Zunke F. (2021). Pathological α-syn aggregation is mediated by glycosphingolipid chain length and the physiological state of α-syn *in vivo* . Proc. Natl. Acad. Sci. U. S. A. 118, e2108489118. 10.1073/pnas.2108489118 34893541 PMC8685670

[B54] Galper J. Dean N. J. Pickford R. Lewis S. J. G. Halliday G. M. Kim W. S. (2022). Lipid pathway dysfunction is prevalent in patients with Parkinson's disease. Brain 145, 3472–3487. 10.1093/brain/awac176 35551349 PMC9586542

[B55] Galvagnion C. Buell A. K. Meisl G. Michaels T. C. Vendruscolo M. Knowles T. P. (2015). Lipid vesicles trigger α-synuclein aggregation by stimulating primary nucleation. Nat. Chem. Biol. 11, 229–234. 10.1038/nchembio.1750 25643172 PMC5019199

[B56] Gegg M. E. Menozzi E. Schapira A. H. V. (2022). Glucocerebrosidase-associated Parkinson disease: pathogenic mechanisms and potential drug treatments. Neurobiol. Dis. 166, 105663. 10.1016/j.nbd.2022.105663 35183702

[B57] Georgas K. Rumballe B. Valerius M. T. Chiu H. S. Thiagarajan R. D. Lesieur E. (2009). Analysis of early nephron patterning reveals a role for distal RV proliferation in fusion to the ureteric tip via a cap mesenchyme-derived connecting segment. Dev. Biol. 332, 273–286. 10.1016/j.ydbio.2009.05.578 19501082

[B58] George A. A. Hayden S. Stanton G. R. Brockerhoff S. E. (2016). Arf6 and the 5'phosphatase of synaptojanin 1 regulate autophagy in cone photoreceptors. Bioessays 38 (Suppl. 1), S119–S135. 10.1002/bies.201670913 27417116

[B59] George J. M. Jin H. Woods W. S. Clayton D. F. (1995). Characterization of a novel protein regulated during the critical period for song learning in the zebra finch. Neuron 15, 361–372. 10.1016/0896-6273(95)90040-3 7646890

[B60] Ghosh D. Mehra S. Sahay S. Singh P. K. Maji S. K. (2017). α-synuclein aggregation and its modulation. Int. J. Biol. Macromol. 100, 37–54. 10.1016/j.ijbiomac.2016.10.021 27737778

[B61] Goedert M. Masuda-Suzukake M. Falcon B. (2017). Like prions: the propagation of aggregated tau and α-synuclein in neurodegeneration. Brain 140, 266–278. 10.1093/brain/aww230 27658420

[B62] Grey M. Linse S. Nilsson H. Brundin P. Sparr E. (2011). Membrane interaction of α-synuclein in different aggregation states. J. Park. Dis. 1, 359–371. 10.3233/JPD-2011-11067 23933657

[B63] Hadaczek P. Wu G. Sharma N. Ciesielska A. Bankiewicz K. Davidow A. L. (2015). GDNF signaling implemented by GM1 ganglioside; failure in Parkinson's disease and GM1-deficient murine model. Exp. Neurol. 263, 177–189. 10.1016/j.expneurol.2014.10.010 25448159

[B64] Hamilton J. A. Hillard C. J. Spector A. A. Watkins P. A. (2007). Brain uptake and utilization of fatty acids, lipids and lipoproteins: application to neurological disorders. J. Mol. Neurosci. 33, 2–11. 10.1007/s12031-007-0060-1 17901539

[B65] Hasegawa T. Konno M. Baba T. Sugeno N. Kikuchi A. Kobayashi M. (2011). The AAA-ATPase VPS4 regulates extracellular secretion and lysosomal targeting of α-synuclein. PloS one 6, e29460. 10.1371/journal.pone.0029460 22216284 PMC3245276

[B66] Hsu L. J. Mallory M. Xia Y. Veinbergs I. Hashimoto M. Yoshimoto M. (1998). Expression pattern of synucleins (non-Abeta component of Alzheimer's disease amyloid precursor protein/alpha-synuclein) during murine brain development. J. Neurochem. 71, 338–344. 10.1046/j.1471-4159.1998.71010338.x 9648883

[B67] Huang M. Wang B. Li X. Fu C. Wang C. Kang X. (2019). α-Synuclein: a multifunctional player in exocytosis, endocytosis, and vesicle recycling. Front. Neurosci. 13, 28. 10.3389/fnins.2019.00028 30745863 PMC6360911

[B68] Jacob R. S. Eichmann C. Dema A. Mercadante D. Selenko P. (2021a). α-Synuclein plasma membrane localization correlates with cellular phosphatidylinositol polyphosphate levels. eLife 10, e61951. 10.7554/eLife.61951 33587036 PMC7929559

[B69] Jacob R. S. Eichmann C. Dema A. Mercadante D. Selenko P. (2021b). α-Synuclein plasma membrane localization correlates with cellular phosphatidylinositol polyphosphate levels. Elife 10, e61951. 10.7554/eLife.61951 33587036 PMC7929559

[B70] Jellinger K. A. Korczyn A. D. (2018). Are dementia with Lewy bodies and Parkinson's disease dementia the same disease? BMC Med. 16, 34. 10.1186/s12916-018-1016-8 29510692 PMC5840831

[B71] Jensen P. H. Hager H. Nielsen M. S. Højrup P. Gliemann J. Jakes R. (1999). alpha-synuclein binds to Tau and stimulates the protein kinase A-catalyzed tau phosphorylation of serine residues 262 and 356. J. Biol. Chem. 274, 25481–25489. 10.1074/jbc.274.36.25481 10464279

[B72] Jiang K. Rocha S. Westling A. Kesarimangalam S. Dorfman K. D. Wittung-Stafshede P. (2018). Alpha-synuclein modulates the physical properties of DNA. Chemistry 24, 15685–15690. 10.1002/chem.201803933 30102440 PMC6217799

[B73] Jin Y. Li F. Sonoustoun B. Kondru N. C. Martens Y. A. Qiao W. (2022). APOE4 exacerbates α-synuclein seeding activity and contributes to neurotoxicity in Alzheimer's disease with Lewy body pathology. Acta Neuropathol. 143, 641–662. 10.1007/s00401-022-02421-8 35471463 PMC9107450

[B74] Johnson P. H. Weinreb N. J. Cloyd J. C. Tuite P. J. Kartha R. V. (2020). GBA1 mutations: prospects for exosomal biomarkers in α-synuclein pathologies. Mol. Genet. Metab. 129, 35–46. 10.1016/j.ymgme.2019.10.006 31761523 PMC7002237

[B75] Kalia L. V. Lang A. E. (2015). Parkinson's disease. Lancet 386, 896–912. 10.1016/s0140-6736(14)61393-3 25904081

[B76] Khounlo R. Hawk B. J. D. Khu T.-M. Yoo G. Lee N. K. Pierson J. (2021). Membrane binding of α-synuclein stimulates expansion of SNARE-dependent fusion pore. Front. Cell Dev. Biol. 9, 663431. 10.3389/fcell.2021.663431 34350173 PMC8326570

[B77] Kontopoulos E. Parvin J. D. Feany M. B. (2006). Alpha-synuclein acts in the nucleus to inhibit histone acetylation and promote neurotoxicity. Hum. Mol. Genet. 15, 3012–3023. 10.1093/hmg/ddl243 16959795

[B78] Krebs C. E. Karkheiran S. Powell J. C. Cao M. Makarov V. Darvish H. (2013). The Sac1 domain of *<scp>SYNJ</scp> 1* identified mutated in a family with early‐onset progressive P arkinsonism with generalized seizures. Hum. Mutat. 34, 1200–1207. 10.1002/humu.22372 23804563 PMC3790461

[B79] Krumova P. Meulmeester E. Garrido M. Tirard M. Hsiao H. H. Bossis G. (2011). Sumoylation inhibits alpha-synuclein aggregation and toxicity. J. Cell Biol. 194, 49–60. 10.1083/jcb.201010117 21746851 PMC3135405

[B80] Lee H. J. Kang S. J. Lee K. Im H. (2011). Human α-synuclein modulates vesicle trafficking through its interaction with prenylated Rab acceptor protein 1. Biochem. Biophys. Res. Commun. 412, 526–531. 10.1016/j.bbrc.2011.07.028 21798244

[B81] Lee J. Y. Marian O. C. Don A. S. (2021). Defective lysosomal lipid catabolism as a common pathogenic mechanism for dementia. Neuromolecular Med. 23, 1–24. 10.1007/s12017-021-08644-4 33550528

[B82] Lee P. H. Lim T. S. Shin H. W. Yong S. W. Nam H. S. Sohn Y. H. (2009). Serum cholesterol levels and the risk of multiple system atrophy: a case-control study. Mov. Disord. 24, 752–758. 10.1002/mds.22459 19185013

[B83] Lefterov I. Wolfe C. M. Fitz N. F. Nam K. N. Letronne F. Biedrzycki R. J. (2019). APOE2 orchestrated differences in transcriptomic and lipidomic profiles of postmortem AD brain. Alzheimers Res. Ther. 11, 113. 10.1186/s13195-019-0558-0 31888770 PMC6937981

[B84] Li D. Zhang J. Liu Q. (2022). Brain cell type-specific cholesterol metabolism and implications for learning and memory. Trends Neurosci. 45, 401–414. 10.1016/j.tins.2022.01.002 35184896

[B85] Lou X. Kim J. Hawk B. J. Shin Y.-K. (2017). α-Synuclein may cross-bridge v-SNARE and acidic phospholipids to facilitate SNARE-dependent vesicle docking. Biochem. J. 474, 2039–2049. 10.1042/BCJ20170200 28495859 PMC5772654

[B86] Lozupone M. Panza F. (2024). Impact of apolipoprotein E isoforms on sporadic Alzheimer's disease: beyond the role of amyloid beta. Neural Regen. Res. 19, 80–83. 10.4103/1673-5374.375316 37488848 PMC10479857

[B87] Lyra P. Machado V. Rota S. Chaudhuri K. R. Botelho J. Mendes J. J. (2023). Revisiting alpha-synuclein pathways to inflammation. Int. J. Mol. Sci. 24, 7137. 10.3390/ijms24087137 37108299 PMC10138587

[B88] Madureira M. Connor-Robson N. Wade-Martins R. (2020). LRRK2: autophagy and lysosomal activity. Front. Neurosci. 14, 498. 10.3389/fnins.2020.00498 32523507 PMC7262160

[B89] Makasewicz K. Linse S. Sparr E. (2024). Interplay of α-synuclein with lipid membranes: cooperative adsorption, membrane remodeling and coaggregation. JACS Au 4, 1250–1262. 10.1021/jacsau.3c00579 38665673 PMC11040681

[B90] Makasewicz K. Wennmalm S. Stenqvist B. Fornasier M. Andersson A. Jonsson P. (2021). Cooperativity of α-synuclein binding to lipid membranes. ACS Chem. Neurosci. 12, 2099–2109. 10.1021/acschemneuro.1c00006 34076426 PMC8291482

[B91] Man W. K. Tahirbegi B. Vrettas M. D. Preet S. Ying L. Vendruscolo M. (2021). The docking of synaptic vesicles on the presynaptic membrane induced by α-synuclein is modulated by lipid composition. Nat. Commun. 12, 927. 10.1038/s41467-021-21027-4 33568632 PMC7876145

[B92] Manzanza N. O. Sedlackova L. Kalaria R. N. (2021). Alpha-synuclein post-translational modifications: implications for pathogenesis of Lewy body disorders. Front. Aging Neurosci. 13, 690293. 10.3389/fnagi.2021.690293 34248606 PMC8267936

[B93] Maroteaux L. Campanelli J. T. Scheller R. H. (1988). Synuclein: a neuron-specific protein localized to the nucleus and presynaptic nerve terminal. J. Neurosci. official J. Soc. Neurosci. 8, 2804–2815. 10.1523/jneurosci.08-08-02804.1988 PMC6569395 3411354

[B94] Martinez Z. Zhu M. Han S. Fink A. L. (2007). GM1 specifically interacts with alpha-synuclein and inhibits fibrillation. Biochemistry 46, 1868–1877. 10.1021/bi061749a 17253773

[B95] Mazzulli J. R. Zunke F. Tsunemi T. Toker N. J. Jeon S. Burbulla L. F. (2016). Activation of β-glucocerebrosidase reduces pathological α-synuclein and restores lysosomal function in Parkinson's patient midbrain neurons. J. Neurosci. 36, 7693–7706. 10.1523/JNEUROSCI.0628-16.2016 27445146 PMC4951575

[B96] McFarland M. A. Ellis C. E. Markey S. P. Nussbaum R. L. (2008). Proteomics analysis identifies phosphorylation-dependent alpha-synuclein protein interactions. Mol. Cell. Proteomics 7, 2123–2137. 10.1074/mcp.M800116-MCP200 18614564 PMC2577212

[B97] McNamara R. K. Liu Y. Jandacek R. Rider T. Tso P. (2008). The aging human orbitofrontal cortex: decreasing polyunsaturated fatty acid composition and associated increases in lipogenic gene expression and stearoyl-CoA desaturase activity. Prostagl. Leukot. Essent. Fat. Acids 78, 293–304. 10.1016/j.plefa.2008.04.001 PMC2494852 18499418

[B98] Mehra S. Gadhe L. Bera R. Sawner A. S. Maji S. K. (2021). Structural and functional insights into α-synuclein fibril polymorphism. Biomolecules 11, 1419. 10.3390/biom11101419 34680054 PMC8533119

[B99] Menges S. Minakaki G. Schaefer P. M. Meixner H. Prots I. Schlötzer-Schrehardt U. (2017). Alpha-synuclein prevents the formation of spherical mitochondria and apoptosis under oxidative stress. Sci. Rep-Uk 7, 42942. 10.1038/srep42942 PMC5320486 28224980

[B100] Mesa-Herrera F. Taoro-Gonzalez L. Valdes-Baizabal C. Diaz M. Marin R. (2019). Lipid and lipid raft alteration in aging and neurodegenerative diseases: a window for the development of new biomarkers. Int. J. Mol. Sci. 20, 3810. 10.3390/ijms20153810 31382686 PMC6696273

[B101] Middleton E. R. Rhoades E. (2010). Effects of curvature and composition on α-synuclein binding to lipid vesicles. Biophys. J. 99, 2279–2288. 10.1016/j.bpj.2010.07.056 20923663 PMC3042580

[B102] Miraglia F. Ricci A. Rota L. Colla E. (2018). Subcellular localization of alpha-synuclein aggregates and their interaction with membranes. Neural Regen. Res. 13, 1136–1144. 10.4103/1673-5374.235013 30028312 PMC6065224

[B103] Miranda A. M. Ashok A. Chan R. B. Zhou B. Xu Y. McIntire L. B. (2022). Effects of APOE4 allelic dosage on lipidomic signatures in the entorhinal cortex of aged mice. Transl. Psychiatry 12, 129. 10.1038/s41398-022-01881-6 35351864 PMC8964762

[B104] Moors T. E. Maat C. A. Niedieker D. Mona D. Petersen D. Timmermans-Huisman E. (2021). The subcellular arrangement of alpha-synuclein proteoforms in the Parkinson's disease brain as revealed by multicolor STED microscopy. Acta Neuropathol. 142, 423–448. 10.1007/s00401-021-02329-9 34115198 PMC8357756

[B105] Morris A. M. Finke R. G. (2009). Alpha-synuclein aggregation variable temperature and variable pH kinetic data: a re-analysis using the Finke-Watzky 2-step model of nucleation and autocatalytic growth. Biophys. Chem. 140, 9–15. 10.1016/j.bpc.2008.11.003 19101068

[B106] Mota-Martorell N. Andres-Benito P. Martin-Gari M. Galo-Licona J. D. Sol J. Fernandez-Bernal A. (2022). Selective brain regional changes in lipid profile with human aging. Geroscience 44, 763–783. 10.1007/s11357-022-00527-1 35149960 PMC9135931

[B107] Nakamura K. Nemani V. M. Azarbal F. Skibinski G. Levy J. M. Egami K. (2011). Direct membrane association drives mitochondrial fission by the Parkinson disease-associated protein alpha-synuclein. J. Biol. Chem. 286, 20710–20726. 10.1074/jbc.M110.213538 21489994 PMC3121472

[B108] Nalls M. A. Blauwendraat C. Vallerga C. L. Heilbron K. Bandres-Ciga S. Chang D. (2019). Identification of novel risk loci, causal insights, and heritable risk for Parkinson's disease: a meta-analysis of genome-wide association studies. Lancet Neurol. 18, 1091–1102. 10.1016/S1474-4422(19)30320-5 31701892 PMC8422160

[B109] Nalls M. A. Duran R. Lopez G. Kurzawa-Akanbi M. McKeith I. G. Chinnery P. F. (2013). A multicenter study of glucocerebrosidase mutations in dementia with Lewy bodies. JAMA Neurol. 70, 727–735. 10.1001/jamaneurol.2013.1925 23588557 PMC3841974

[B110] Nasaruddin M. L. Holscher C. Kehoe P. Graham S. F. Green B. D. (2016). Wide-ranging alterations in the brain fatty acid complement of subjects with late Alzheimer's disease as detected by GC-MS. Am. J. Transl. Res. 8, 154–165. 27069549 PMC4759425

[B111] Nedelec T. Couvy-Duchesne B. Darves-Bornoz A. Couronne R. Monnet F. Gantzer L. (2023). A comparison between early presentation of dementia with Lewy bodies, alzheimer's disease, and Parkinson's disease: evidence from routine primary care and UK biobank data. Ann. Neurol. 94, 259–270. 10.1002/ana.26670 37098633

[B112] Neumann J. Bras J. Deas E. O'Sullivan S. S. Parkkinen L. Lachmann R. H. (2009). Glucocerebrosidase mutations in clinical and pathologically proven Parkinson's disease. Brain 132, 1783–1794. 10.1093/brain/awp044 19286695 PMC2702833

[B113] Noe E. Marder K. Bell K. L. Jacobs D. M. Manly J. J. Stern Y. (2004). Comparison of dementia with Lewy bodies to Alzheimer's disease and Parkinson's disease with dementia. Mov. Disord. 19, 60–67. 10.1002/mds.10633 14743362

[B114] Nordengen K. Morland C. (2024). From synaptic physiology to synaptic pathology: the enigma of α-synuclein. Int. J. Mol. Sci. 25, 986. 10.3390/ijms25020986 38256059 PMC10815905

[B115] Olgiati S. De Rosa A. Quadri M. Criscuolo C. Breedveld G. J. Picillo M. (2014). PARK20 caused by SYNJ1 homozygous Arg258Gln mutation in a new Italian family. Neurogenetics 15, 183–188. 10.1007/s10048-014-0406-0 24816432

[B116] Orru C. D. Ma T. C. Hughson A. G. Groveman B. R. Srivastava A. Galasko D. (2021). A rapid α-synuclein seed assay of Parkinson's disease CSF panel shows high diagnostic accuracy. Ann. Clin. Transl. Neurol. 8, 374–384. 10.1002/acn3.51280 33373501 PMC7886040

[B117] Oueslati A. Fournier M. Lashuel H. A. (2010). “Chapter 7 - role of post-translational modifications in modulating the structure, function and toxicity of α-synuclein: implications for Parkinson’s disease pathogenesis and therapies,” in Progress in brain research. Editors Björklund A. Cenci M. A. (Elsevier), 115–145. 10.1016/S0079-6123(10)83007-9 20696318

[B118] Outeiro T. F. Koss D. J. Erskine D. Walker L. Kurzawa-Akanbi M. Burn D. (2019). Dementia with Lewy bodies: an update and outlook. Mol. Neurodegener. 14, 5. 10.1186/s13024-019-0306-8 30665447 PMC6341685

[B119] Pan P. Y. Li X. Wang J. Powell J. Wang Q. Zhang Y. (2017). Parkinson's disease-associated LRRK2 hyperactive kinase mutant disrupts synaptic vesicle trafficking in ventral midbrain neurons. J. Neurosci. official J. Soc. Neurosci. 37, 11366–11376. 10.1523/JNEUROSCI.0964-17.2017 PMC5700420 29054882

[B120] Paradies G. Paradies V. De Benedictis V. Ruggiero F. M. Petrosillo G. (2014). Functional role of cardiolipin in mitochondrial bioenergetics. Biochim. Biophys. Acta (BBA) - Bioenerg. 1837, 408–417. 10.1016/j.bbabio.2013.10.006 24183692

[B121] Payton J. E. Perrin R. J. Woods W. S. George J. M. (2004). Structural determinants of PLD2 inhibition by α-synuclein. J. Mol. Biol. 337, 1001–1009. 10.1016/j.jmb.2004.02.014 15033366

[B122] Peng C. Gathagan R. J. Covell D. J. Medellin C. Stieber A. Robinson J. L. (2018). Cellular milieu imparts distinct pathological α-synuclein strains in α-synucleinopathies. Nature 557, 558–563. 10.1038/s41586-018-0104-4 29743672 PMC5970994

[B123] Perera R. M. Zoncu R. Lucast L. De Camilli P. Toomre D. (2006). Two synaptojanin 1 isoforms are recruited to clathrin-coated pits at different stages. Proc. Natl. Acad. Sci. U. S. A. 103, 19332–19337. 10.1073/pnas.0609795104 17158794 PMC1693868

[B124] Pifferi F. Laurent B. Plourde M. (2021). Lipid transport and metabolism at the blood-brain interface: implications in health and disease. Front. Physiol. 12, 645646. 10.3389/fphys.2021.645646 33868013 PMC8044814

[B125] Pifl C. Rajput A. Reither H. Blesa J. Cavada C. Obeso J. A. (2014). Is Parkinson's disease a vesicular dopamine storage disorder? Evidence from a study in isolated synaptic vesicles of human and nonhuman primate striatum. J. Neurosci. 34, 8210–8218. 10.1523/JNEUROSCI.5456-13.2014 24920625 PMC6608236

[B126] Pirc K. Ulrih N. P. (2015). α-Synuclein interactions with phospholipid model membranes: key roles for electrostatic interactions and lipid-bilayer structure. Biochim. Biophys. acta 1848, 2002–2012. 10.1016/j.bbamem.2015.06.021 26119565

[B127] Pires M. Rego A. C. (2023). Apoe4 and alzheimer's disease pathogenesis-mitochondrial deregulation and targeted therapeutic strategies. Int. J. Mol. Sci. 24, 778. 10.3390/ijms24010778 36614219 PMC9821307

[B128] Poewe W. Stankovic I. Halliday G. Meissner W. G. Wenning G. K. Pellecchia M. T. (2022). Multiple system atrophy. Nat. Rev. Dis. Prim. 8, 56. 10.1038/s41572-022-00382-6 36008429

[B129] Poitelon Y. Kopec A. M. Belin S. (2020). Myelin fat facts: an overview of lipids and fatty acid metabolism. Cells 9, 812. 10.3390/cells9040812 32230947 PMC7226731

[B130] Pozo Devoto V. M. Dimopoulos N. Alloatti M. Pardi M. B. Saez T. M. Otero M. G. (2017). αSynuclein control of mitochondrial homeostasis in human-derived neurons is disrupted by mutations associated with Parkinson's disease. Sci. Rep. 7, 5042. 10.1038/s41598-017-05334-9 28698628 PMC5506004

[B131] Prusiner S. B. (2012). Cell biology. A unifying role for prions in neurodegenerative diseases. Science 336, 1511–1513. 10.1126/science.1222951 22723400 PMC3942086

[B132] Quadri M. Fang M. Picillo M. Olgiati S. Breedveld G. J. Graafland J. (2013). Mutation in the SYNJ1 gene associated with autosomal recessive, early-onset Parkinsonism. Hum. Mutat. 34, 1208–1215. 10.1002/humu.22373 23804577

[B133] Ramalingam N. Jin S. X. Moors T. E. Fonseca-Ornelas L. Shimanaka K. Lei S. (2023). Dynamic physiological α-synuclein S129 phosphorylation is driven by neuronal activity. Npj Park. Dis. 9, 4. 10.1038/s41531-023-00444-w PMC9842642 36646701

[B134] Rcom-H'cheo-Gauthier A. N. Osborne S. L. Meedeniya A. C. Pountney D. L. (2016). Calcium: alpha-synuclein interactions in alpha-synucleinopathies. Front. Neurosci. 10, 570. 10.3389/fnins.2016.00570 28066161 PMC5167751

[B135] Robinson J. L. Lee E. B. Xie S. X. Rennert L. Suh E. Bredenberg C. (2018). Neurodegenerative disease concomitant proteinopathies are prevalent, age-related and APOE4-associated. Brain 141, 2181–2193. 10.1093/brain/awy146 29878075 PMC6022546

[B136] Rocha E. M. De Miranda B. Sanders L. H. (2018). Alpha-synuclein: pathology, mitochondrial dysfunction and neuroinflammation in Parkinson's disease. Neurobiol. Dis. 109, 249–257. 10.1016/j.nbd.2017.04.004 28400134

[B137] Rodriguez J. A. Ivanova M. I. Sawaya M. R. Cascio D. Reyes F. E. Shi D. (2015). Structure of the toxic core of α-synuclein from invisible crystals. Nature 525, 486–490. 10.1038/nature15368 26352473 PMC4791177

[B138] Roeters S. J. Strunge K. Pedersen K. B. Golbek T. W. Bregnhøj M. Zhang Y. (2023). Elevated concentrations cause upright alpha-synuclein conformation at lipid interfaces. Nat. Commun. 14, 5731. 10.1038/s41467-023-39843-1 37723164 PMC10507035

[B139] Rouser G. Yamamoto A. (1968). Curvilinear regression course of human brain lipid composition changes with age. Lipids 3, 284–287. 10.1007/BF02531202 17805871

[B140] Roy S. Winton M. J. Black M. M. Trojanowski J. Q. Lee V. M. (2007). Rapid and intermittent cotransport of slow component-b proteins. J. Neurosci. official J. Soc. Neurosci. 27, 3131–3138. 10.1523/JNEUROSCI.4999-06.2007 PMC6672457 17376974

[B141] Rutledge J. Lehallier B. Zarifkar P. Losada P. M. Shahid-Besanti M. Western D. (2024). Comprehensive proteomics of CSF, plasma, and urine identify DDC and other biomarkers of early Parkinson's disease. Acta Neuropathol. 147, 52. 10.1007/s00401-024-02706-0 38467937 PMC10927779

[B142] Ryan T. Bamm V. V. Stykel M. G. Coackley C. L. Humphries K. M. Jamieson-Williams R. (2018). Cardiolipin exposure on the outer mitochondrial membrane modulates α-synuclein. Nat. Commun. 9, 817. 10.1038/s41467-018-03241-9 29483518 PMC5827019

[B143] Ryu S. Baek I. Liew H. (2019). Sumoylated α-synuclein translocates into the nucleus by karyopherin α6. Mol. Cell. Toxicol. 15, 103–109. 10.1007/s13273-019-0012-1

[B144] Sahoo S. Padhy A. A. Kumari V. Mishra P. (2022). Role of ubiquitin-proteasome and autophagy-lysosome pathways in α-synuclein aggregate clearance. Mol. Neurobiol. 59, 5379–5407. 10.1007/s12035-022-02897-1 35699874

[B145] Saxton W. M. Hollenbeck P. J. (2012). The axonal transport of mitochondria. J. Cell Sci. 125, 2095–2104. 10.1242/jcs.053850 22619228 PMC3656622

[B146] Schechter M. Sharon R. (2021). An emerging role for phosphoinositides in the pathophysiology of Parkinson's disease. J. Park. Dis. 11, 1725–1750. 10.3233/JPD-212684 PMC8609718 34151859

[B147] Schneider Y. Gauer C. Andert M. Hoffmann A. Riemenschneider M. J. Krebs W. (2024). Distinct forebrain regions define a dichotomous astrocytic profile in multiple system atrophy. Acta Neuropathol. Commun. 12, 1. 10.1186/s40478-023-01699-3 38167307 PMC10759635

[B148] Schweighauser M. Shi Y. Tarutani A. Kametani F. Murzin A. G. Ghetti B. (2020). Structures of α-synuclein filaments from multiple system atrophy. Nature 585, 464–469. 10.1038/s41586-020-2317-6 32461689 PMC7116528

[B149] Segrest J. P. De Loof H. Dohlman J. G. Brouillette C. G. Anantharamaiah G. M. (1990). Amphipathic helix motif: classes and properties. Proteins Struct. Funct. Bioinforma. 8, 103–117. 10.1002/prot.340080202 2235991

[B150] Shahmoradian S. H. Lewis A. J. Genoud C. Hench J. Moors T. E. Navarro P. P. (2019). Lewy pathology in Parkinson's disease consists of crowded organelles and lipid membranes. Nat. Neurosci. 22, 1099–1109. 10.1038/s41593-019-0423-2 31235907

[B151] Shahnawaz M. Mukherjee A. Pritzkow S. Mendez N. Rabadia P. Liu X. (2020). Discriminating α-synuclein strains in Parkinson's disease and multiple system atrophy. Nature 578, 273–277. 10.1038/s41586-020-1984-7 32025029 PMC7066875

[B152] Sharma M. Burré J. (2023). α-Synuclein in synaptic function and dysfunction. Trends Neurosci. 46, 153–166. 10.1016/j.tins.2022.11.007 36567199 PMC9877183

[B153] Shvadchak V. V. Yushchenko D. A. Pievo R. Jovin T. M. (2011). The mode of α-synuclein binding to membranes depends on lipid composition and lipid to protein ratio. FEBS Lett. 585, 3513–3519. 10.1016/j.febslet.2011.10.006 22004764

[B154] So R. W. L. Watts J. C. (2023). α-Synuclein conformational strains as drivers of phenotypic heterogeneity in neurodegenerative diseases. J. Mol. Biol. 435, 168011. 10.1016/j.jmb.2023.168011 36792008

[B155] Soper J. H. Kehm V. Burd C. G. Bankaitis V. A. Lee V. M. Y. (2011). Aggregation of α-synuclein in *S. cerevisiae* is associated with defects in endosomal trafficking and phospholipid biosynthesis. J. Mol. Neurosci. 43, 391–405. 10.1007/s12031-010-9455-5 20890676 PMC3147281

[B156] Spillantini M. G. Crowther R. A. Jakes R. Cairns N. J. Lantos P. L. Goedert M. (1998). Filamentous alpha-synuclein inclusions link multiple system atrophy with Parkinson's disease and dementia with Lewy bodies. Neurosci. Lett. 251, 205–208. 10.1016/s0304-3940(98)00504-7 9726379

[B157] Stoker T. B. Barker R. A. (2020). Recent developments in the treatment of Parkinson's Disease. F1000Res 9, 862. 10.12688/f1000research.25634.1 PMC7400683 32789002

[B158] Sugeno N. Jäckel S. Voigt A. Wassouf Z. Schulze-Hentrich J. Kahle P. J. (2016). α-Synuclein enhances histone H3 lysine-9 dimethylation and H3K9me2-dependent transcriptional responses. Sci. Rep. 6, 36328. 10.1038/srep36328 27808254 PMC5093762

[B159] Surguchov A. (2023). α-Synuclein and mechanisms of epigenetic regulation. Brain Sci. 13, 150. 10.3390/brainsci13010150 36672131 PMC9857298

[B160] Svennerholm L. Bostrom K. Helander C. G. Jungbjer B. (1991). Membrane lipids in the aging human brain. J. Neurochem. 56, 2051–2059. 10.1111/j.1471-4159.1991.tb03466.x 2027013

[B161] Taghavi S. Chaouni R. Tafakhori A. Azcona L. J. Firouzabadi S. G. Omrani M. D. (2018). A clinical and molecular genetic study of 50 families with autosomal recessive parkinsonism revealed known and novel gene mutations. Mol. Neurobiol. 55, 3477–3489. 10.1007/s12035-017-0535-1 28502045 PMC5683945

[B162] Taguchi K. Watanabe Y. Tsujimura A. Tanaka M. (2016). Brain region-dependent differential expression of alpha-synuclein. J. Comp. Neurol. 524, 1236–1258. 10.1002/cne.23901 26358191

[B163] Tang Y. Das U. Scott D. A. Roy S. (2012). The slow axonal transport of alpha-synuclein--mechanistic commonalities amongst diverse cytosolic cargoes. Cytoskelet. Hob. 69, 506–513. 10.1002/cm.21019 PMC3376189 22344896

[B164] Teixeira M. Sheta R. Idi W. Oueslati A. (2021). Alpha-synuclein and the endolysosomal system in Parkinson's disease: guilty by association. Biomolecules 11, 1333. 10.3390/biom11091333 34572546 PMC8472725

[B165] Thayanidhi N. Helm J. R. Nycz D. C. Bentley M. Liang Y. Hay J. C. (2010). Alpha-synuclein delays endoplasmic reticulum (ER)-to-Golgi transport in mammalian cells by antagonizing ER/Golgi SNAREs. Mol. Biol. Cell 21, 1850–1863. 10.1091/mbc.e09-09-0801 20392839 PMC2877643

[B166] Timney B. L. Raveh B. Mironska R. Trivedi J. M. Kim S. J. Russel D. (2016). Simple rules for passive diffusion through the nuclear pore complex. J. Cell Biol. 215, 57–76. 10.1083/jcb.201601004 27697925 PMC5057280

[B167] Torre-Muruzabal T. Van der Perren A. Coens A. Gelders G. Janer A. B. Camacho-Garcia S. (2023). Host oligodendrogliopathy and α-synuclein strains dictate disease severity in multiple system atrophy. Brain 146, 237–251. 10.1093/brain/awac061 35170728

[B168] Tsakiri M. Tsichlis I. Zivko C. Demetzos C. Mahairaki V. (2024). Lipidic nanoparticles, extracellular vesicles and hybrid platforms as advanced medicinal products: future therapeutic prospects for neurodegenerative diseases. Pharmaceutics 16, 350. 10.3390/pharmaceutics16030350 38543244 PMC10975844

[B169] Tsuang D. Leverenz J. B. Lopez O. L. Hamilton R. L. Bennett D. A. Schneider J. A. (2013). APOE ε4 increases risk for dementia in pure synucleinopathies. JAMA Neurol. 70, 223–228. 10.1001/jamaneurol.2013.600 23407718 PMC3580799

[B170] Tuttle M. D. Comellas G. Nieuwkoop A. J. Covell D. J. Berthold D. A. Kloepper K. D. (2016). Solid-state NMR structure of a pathogenic fibril of full-length human α-synuclein. Nat. Struct. Mol. Biol. 23, 409–415. 10.1038/nsmb.3194 27018801 PMC5034296

[B171] Ueda K. Fukushima H. Masliah E. Xia Y. Iwai A. Yoshimoto M. (1993). Molecular cloning of cDNA encoding an unrecognized component of amyloid in Alzheimer disease. Proc. Natl. Acad. Sci. U. S. A. 90, 11282–11286. 10.1073/pnas.90.23.11282 8248242 PMC47966

[B172] Vanhauwaert R. Kuenen S. Masius R. Bademosi A. Manetsberger J. Schoovaerts N. (2017). The SAC1 domain in synaptojanin is required for autophagosome maturation at presynaptic terminals. EMBO J. 36, 1392–1411. 10.15252/embj.201695773 28331029 PMC5430236

[B173] Vos M. Dulovic-Mahlow M. Mandik F. Frese L. Kanana Y. Haissatou Diaw S. (2021). Ceramide accumulation induces mitophagy and impairs β-oxidation in PINK1 deficiency. Proc. Natl. Acad. Sci. U. S. A. 118, e2025347118. 10.1073/pnas.2025347118 34686591 PMC8639384

[B174] Walker L. C. Jucker M. (2015). Neurodegenerative diseases: expanding the prion concept. Annu. Rev. Neurosci. 38, 87–103. 10.1146/annurev-neuro-071714-033828 25840008 PMC4803040

[B175] Wang R. Sun H. Ren H. Wang G. (2020). α-Synuclein aggregation and transmission in Parkinson's disease: a link to mitochondria and lysosome. Sci. China Life Sci. 63, 1850–1859. 10.1007/s11427-020-1756-9 32681494

[B176] Watanabe S. Mamer L. E. Raychaudhuri S. Luvsanjav D. Eisen J. Trimbuch T. (2018). Synaptojanin and endophilin mediate neck formation during ultrafast endocytosis. Neuron 98, 1184–1197 e6. 10.1016/j.neuron.2018.06.005 29953872 PMC6086574

[B177] Wei J. Wong L. C. Boland S. (2023). Lipids as emerging biomarkers in neurodegenerative diseases. Int. J. Mol. Sci. 25, 131. 10.3390/ijms25010131 38203300 PMC10778656

[B178] Westphal C. H. Chandra S. S. (2013). Monomeric synucleins generate membrane curvature. J. Biol. Chem. 288, 1829–1840. 10.1074/jbc.M112.418871 23184946 PMC3548493

[B179] Wiseman J. A. Murray H. C. Faull R. Dragunow M. Turner C. P. Dieriks B. V. (2024). Aggregate-prone brain regions in Parkinson’s disease are rich in unique N-terminus α-synuclein conformers with high proteolysis susceptibility. NPJ Park. Dis. 10, 1. 10.1038/s41531-023-00614-w PMC10762179 38167744

[B180] Woerman A. L. Oehler A. Kazmi S. A. Lee J. Halliday G. M. Middleton L. T. (2019). Multiple system atrophy prions retain strain specificity after serial propagation in two different Tg(SNCA*A53T) mouse lines. Acta Neuropathol. 137, 437–454. 10.1007/s00401-019-01959-4 30690664 PMC6454887

[B181] Wood P. L. Tippireddy S. Feriante J. Woltjer R. L. (2018). Augmented frontal cortex diacylglycerol levels in Parkinson's disease and Lewy Body Disease. PloS one 13, e0191815. 10.1371/journal.pone.0191815 29513680 PMC5841652

[B182] Wu G. Lu Z. H. Kulkarni N. Amin R. Ledeen R. W. (2011). Mice lacking major brain gangliosides develop parkinsonism. Neurochem. Res. 36, 1706–1714. 10.1007/s11064-011-0437-y 21399908 PMC3155038

[B183] Xiong H. Callaghan D. Jones A. Walker D. G. Lue L. F. Beach T. G. (2008). Cholesterol retention in Alzheimer's brain is responsible for high beta- and gamma-secretase activities and Abeta production. Neurobiol. Dis. 29, 422–437. 10.1016/j.nbd.2007.10.005 18086530 PMC2720683

[B184] Xu L. Nussinov R. Ma B. (2016). Coupling of the non-amyloid-component (NAC) domain and the KTK(E/Q)GV repeats stabilize the α-synuclein fibrils. Eur. J. Med. Chem. 121, 841–850. 10.1016/j.ejmech.2016.01.044 26873872 PMC4960003

[B185] Yamazaki Y. Zhao N. Caulfield T. R. Liu C. C. Bu G. (2019). Apolipoprotein E and Alzheimer disease: pathobiology and targeting strategies. Nat. Rev. Neurol. 15, 501–518. 10.1038/s41582-019-0228-7 31367008 PMC7055192

[B186] Yang Y. Shi Y. Schweighauser M. Zhang X. Kotecha A. Murzin A. G. (2022). Structures of α-synuclein filaments from human brains with Lewy pathology. Nature 610, 791–795. 10.1038/s41586-022-05319-3 36108674 PMC7613749

[B187] Yin F. (2023). Lipid metabolism and Alzheimer's disease: clinical evidence, mechanistic link and therapeutic promise. FEBS J. 290, 1420–1453. 10.1111/febs.16344 34997690 PMC9259766

[B188] Yoo G. Shin Y.-K. Lee N. K. (2023). The role of α-synuclein in SNARE-mediated synaptic vesicle fusion. J. Mol. Biol. 435, 167775. 10.1016/j.jmb.2022.167775 35931109

[B189] Yu Q. He Z. Zubkov D. Huang S. Kurochkin I. Yang X. (2020). Lipidome alterations in human prefrontal cortex during development, aging, and cognitive disorders. Mol. Psychiatry 25, 2952–2969. 10.1038/s41380-018-0200-8 30089790 PMC7577858

[B190] Zhang J. M. Li X. P. Li J. D. (2019). The roles of post-translational modifications on α-synuclein in the pathogenesis of Parkinson's diseases. Front. Neurosci. 13, 381. 10.3389/fnins.2019.00381 31057362 PMC6482271

[B191] Zhu L. Zhong M. Elder G. A. Sano M. Holtzman D. M. Gandy S. (2015). Phospholipid dysregulation contributes to ApoE4-associated cognitive deficits in Alzheimer's disease pathogenesis. Proc. Natl. Acad. Sci. U. S. A. 112, 11965–11970. 10.1073/pnas.1510011112 26372964 PMC4586834

[B192] Zhu M. Fink A. L. (2003). Lipid binding inhibits alpha-synuclein fibril formation. J. Biol. Chem. 278, 16873–16877. 10.1074/jbc.M210136200 12621030

